# Metagenomic characterization of the metabolism, evolution, and global distribution of *Candidatus* Accumulibacter members in wastewater treatment plants

**DOI:** 10.1093/ismejo/wraf278

**Published:** 2025-12-23

**Authors:** Xiaojing Xie, Liping Chen, Jing Yuan, Haixin Zheng, Lanying Zhang, Xiaokai Yu, Xianghui Liu, Chaohai Wei, Guanglei Qiu

**Affiliations:** School of Environment and Energy, South China University of Technology, Guangzhou, Guangdong, 510006, China; School of Environment and Energy, South China University of Technology, Guangzhou, Guangdong, 510006, China; School of Environment and Energy, South China University of Technology, Guangzhou, Guangdong, 510006, China; School of Environment and Energy, South China University of Technology, Guangzhou, Guangdong, 510006, China; School of Environment and Energy, South China University of Technology, Guangzhou, Guangdong, 510006, China; School of Environment and Energy, South China University of Technology, Guangzhou, Guangdong, 510006, China; Singapore Centre for Environmental Life Sciences Engineering, Nanyang Technological University, Singapore, 637551, Singapore; School of Environment and Energy, South China University of Technology, Guangzhou, Guangdong, 510006, China; The Key Laboratory of Pollution Control and Ecosystem Restoration in Industry Clusters, Ministry of Education, Guangzhou, Guangdong, 510006, China; School of Environment and Energy, South China University of Technology, Guangzhou, Guangdong, 510006, China; Singapore Centre for Environmental Life Sciences Engineering, Nanyang Technological University, Singapore, 637551, Singapore; The Key Laboratory of Pollution Control and Ecosystem Restoration in Industry Clusters, Ministry of Education, Guangzhou, Guangdong, 510006, China; Guangdong Provincial Key Laboratory of Solid Wastes Pollution Control and Recycling, Guangzhou, Guangdong, 510006, China; National Joint Research Center for Ecological Conservation and High Quality Development of the Yellow River Basin, Beijing, 100012, China

**Keywords:** wastewater treatment plants, *Candidatus* Accumulibacter, phylogeny, evolutionary characteristics, metabolic potential

## Abstract

Deciphering the genomic basis of ecological diversification in activated sludge microbiomes is essential for optimizing treatment technology and advancing microbial ecology. Here, we present a global genome-resolved investigation of *Candidatus* Accumulibacter, the primary functional agent of enhanced biological phosphorus removal, based on 828 metagenomes from wastewater treatment plants across six continents. We recovered 104 high-quality *Candidatus* Accumulibacter metagenome-assembled genomes, discovering a new clade (Clade IV), substantially expanding the known phylogenetic diversity and revealing a ubiquitous yet geographically heterogeneous global distribution. Phylogenomic and pangenome analyses uncovered extensive clade-specific gene gain and loss, particularly in nitrogen metabolism, suggesting divergent evolutionary trajectories shaped by relaxed selection and niche adaptation. Genome-wide patterns of convergent streamlining and enriched antiviral defense systems indicate selective pressures from strong competition and viral predation. Constraint-based metabolic modeling revealed pervasive amino acid autotrophies and metabolic complementarity, coupled with distinct carbon utilization strategies that support ecological specialization across operational settings. Experimental validation reconciled model-phenotype discrepancies, highlighting the importance of transporter promiscuity and gene regulation in carbon substrate assimilation. Collectively, our findings redefine *Candidatus* Accumulibacter as a dynamic model of microbial genome plasticity, metabolic adaptation, and ecological resilience, providing an insight into understanding how microbial communities adapt and respond under engineered environmental conditions.

## Introduction

Phosphorus (P) removal from wastewater is an essential step in wastewater treatment to prevent environmental impacts to receiving water bodies (e.g. eutrophication) [1]. Among the various treatment strategies, enhanced biological phosphorus removal (EBPR) processes have been widely implemented in wastewater treatment plants (WWTPs) due to their high efficiency and low energy demand, which rely on phosphate-accumulating organisms (PAOs) to mediate effective phosphorus capture and removal [2–4].

Within the diverse PAO community, *Candidatus* Accumulibacter has emerged as both the primary functional agent driving EBPR and a globally dominant member of the activated sludge microbial communities [5]. This organism utilizes intracellular polyphosphate (poly-P) as an energy source to assimilate volatile fatty acids (VFAs) and other simple carbon substrates under anaerobic conditions, storing them as intracellular polymers [6–9]. During subsequent aerobic phases, these storage compounds are oxidized to generate ATP for growth and metabolism, concurrently facilitating phosphate uptake and poly-P re-synthesis [10]. Beyond its classical functional role, *Ca*. Accumulibacter exhibits remarkable metabolic heterogeneity and ecological versatility, suggesting complex adaptive strategies beyond P removal. Deciphering the diversity, metabolic potential, and ecological strategies of *Ca*. Accumulibacter is therefore critical not only for optimizing P removal performance, but for providing a representative model to understand how microbes maintain functional stability via genomic plasticity and metabolic adaptation in human-influenced environments.

With the advances in molecular biology, a phylogenetic framework based on polyphosphate kinase (*ppk1*) genes has been widely adopted, classifying *Ca*. Accumulibacter into two major types (Clade I and Clade II) and several sub-clades (e.g. IA-IH, IIA-IIK) [4, 11–15]. Building upon this framework, numerous studies have attempted to associate specific lineages with distinct metabolic phenotypes, such as linking Clade I members to denitrification potential [15] and Clade II members to carbon source flexibility and glycogen-driven metabolism [14]. These early efforts provided essential foundations for predicting EBPR functionality based on the sub-lineage community structure of *Ca*. Accumulibacter. However, growing genomic and physiological evidence reveals that existing phylogenetic classifications do not consistently predict metabolic capabilities [11]. Even within the same clade, high variability in critical metabolic traits such as nitrate reduction and carbon substrate utilization were observed [8, 16]. This phenomenon of ``intra-clade metabolic heterogeneity'' implies the complexity and dynamism of *Ca*. Accumulibacter's ecological strategies, shaped by frequent gene acquisition, loss, and functional reshaping, necessitating a higher-resolution reassessment of their diversity and adaptive potential.

Compounding these challenges, conventional molecular techniques also present significant limitations. Widely used *ppk1* and 16S rRNA gene primers exhibit systematic biases and coverage blind spots, leading to possible underrepresentation or omission of specific *Ca*. Accumulibacter lineages [17, 18]. For example, commonly employed *ppk1* primers (254F) show 3′-end mismatches with Clade III lineage sequences [18]. Widely used 16S rRNA gene primers fail to comprehensively cover all *Ca*. Accumulibacter members (e.g. in MiDAS and SILVA databases) [17]. Although global 16S rRNA gene datasets such as MiDAS have provided insights into *Ca*. Accumulibacter's biogeography [3, 19], amplification biases and off-target amplification undermine the reliability and completeness of current diversity assessments. Consequently, the understanding of *Ca*. Accumulibacter's true diversity, metabolic potential, and ecological expansion mechanisms remains fragmented and potentially biased, limiting both theoretical advances in EBPR research and broader insights into microbial community evolution in the engineered environment.

Here, we collected activated sludge samples from 81 WWTPs across 31 provinces in China for metagenomic analysis and assembly, and integratively analyzed 747 genome-resolved metagenomic data from global WWTPs across six continents, recovering 104 high-quality *Ca*. Accumulibacter MAGs, discovering a globally distributed new clade (Clade IV). This global dataset significantly expands the phylogenetic diversity of *Ca*. Accumulibacter, resolves numerous uncharacterized lineages, and links genomic traits to ecological distribution patterns. Comparative analyses revealed distinct evolutionary trajectories among clades, as well as pronounced differences in carbon source utilization capacities and amino acid deficiencies, indicating strong metabolic interdependencies within microbial communities. We further characterized their antiviral defense mechanisms and their interactions with viruses, highlighting the potential role in shaping community structure and function. Together, these findings redefine the ecological role and adaptive strategies of this globally dominant PAO and provide a basis for understanding microbial evolution, functional diversification, and community assembly in engineered ecosystems.

## Materials and methods

### Sample collection

In this study, we sampled and sequenced activated sludge from 81 WWTPs across 40 cities from 31 provinces in China and integrated 747 activated sludge metagenomes from National Center for Biotechnology Information (NCBI) Sequence Read Archive (https://ncbi.nlm.nih.gov/sra/), constituting a global activated sludge dataset including 828 metagenomes of activated sludge from WWTPs distributed in six continents across the world. Metagenome-assembled genomes (MAGs) were recovered from those short-read metagenomic data using a modified and standardized assembly, binning, and quality control pipeline (see supplementary information (SI) for details).

### Construction of the *Ca*. Accumulibacter genome dataset

All the raw metagenomic results were processed for quality check and filtration to acquire clean reads using fastp (v0.23.4) with parameters -q 30 -l 50 [20]. Quality-filtered metagenomic reads were assembled into contigs using metaSPAdes (v3.15.5) using default parameters [21]. Reads were mapped back to the MAGs using Bowtie2 (v2.5.1) for relative abundance calculations [22]. SAMtools (v1.2) [23] was used to generate sequence alignment/map format (BAM files). Contig coverage was calculated using the jgi_summarize_bam_contig_depths script from MetaBAT2 (v2.15) [24]. Contig binning was performed with MetaBAT2 with the -minContig 1500 option. The completeness and contamination of bins were assessed using CheckM (v.1.2.3) [25]. Only MAGs with completeness >50% and contamination <10% were retained, following the MIMAG (Minimum Information about a Metagenome-Assembled Genome) standard. Taxonomic assignment was performed using the Genome Taxonomy Database Toolkit (GTDB-Tk v2.3.2), with the GTDB release 214 reference database [26].

To construct a comprehensive high-quality (completeness >90%, contamination <5%) genome dataset for *Ca*. Accumulibacter, 104 high-quality MAGs were recovered from global WWTPs in this study, along with 7 high-quality MAGs newly retrieved from our lab-scale bioreactors (operational parameters are provided in the SI). In addition, we retrieved 94 publicly available high-quality *Ca*. Accumulibacter MAGs from NCBI (accessed in October 2024). These high-quality MAGs were dereplicated using dRep (v3.2.2) [27] based on a minimum alignment fraction of 85% and an average nucleotide identity (ANI) threshold of 99%, selecting the MAG with the highest quality score (QS = completeness – 5 × contamination) from each cluster, resulting in a final set of 136 high-quality, non-redundant *Ca*. Accumulibacter MAGs. Genome annotation was performed using PROKKA (v1.14.5) [28] with default parameters. To evaluate the effectiveness of existing *ppk1*-targeting primers (254F and 1376R [29]) in capturing the *ppk1* diversity of *Ca*. Accumulibacter, all *ppk1* gene sequences (identified based on the PROKKA annotations) extracted from the reconstructed MAGs were aligned against the primer sequences using BLASTn (v2.16.0). Mismatch profiles at primer-binding regions were analyzed to assess potential amplification biases.

### Phylogenomic analysis and metabolic reconstruction

Homologous gene clusters were identified using Panaroo (v1.5.2) with parameters -f 0.7, -c 0.7, -len_dif_percent 0.75, -search_radius 1000 [30]. To explore genome compositional patterns among clades, we defined core genes (present in >80% MAGs) [31] and clade-specific genes (present exclusively in >75% members of a given clade but absent in other *Ca*. Accumulibacter MAGs) [32]. We reconstructed phylogenomic trees based on both *ppk1* and single-copy marker genes. *ppk1* sequences were extracted based on genome annotations generated by PROKKA (v1.14.5) [28]. Single-copy bacterial marker genes were identified and extracted using GTDB-Tk (v2.3.2). *ppk1* sequences and each single-copy gene were aligned by using MAFFT (v7.520) [33]. Poorly aligned positions were trimmed using trimAl (v1.5. rev0, https://github.com/inab/trimal). The trimmed single-copy gene alignments were concatenated into a single supermatrix. Maximum-likelihood phylogenomic trees were inferred using IQ-TREE2 (v2.4.0) [34] with 1000 ultrafast bootstrap replicates and the best-fit substitution model which was selected by using ModelFinder.

Gene gain and loss dynamics were inferred with Count (v10.04) based on the homologous gene family abundance matrix [35]. To enhance sensitivity, Wagner parsimony with a penalty value of 2 was applied. Genes acquired at the last common ancestor (LCA) of *Ca*. Accumulibacter were defined as ancestral genes, whereas those gained after the LCA were considered as derived genes.

ANI values between MAGs were calculated using FastANI (v1.33) [36]. The percentage of conserved proteins (POCP) was determined using POCP-nf [37]. The whole-genome amino acid compositions were assessed with CompareM (v0.1.2) under default parameters (https://github.com/dparks1134/CompareM). Metabolic pathway reconstructions and biochemical potential profiling were conducted using gapseq (v1.0) [38] and METABOLIC (v4.0) [39]. Antiviral defense systems (DSs) were identified with DefenseFinder (v2.0.0) [40]. Only complete defense system (DS) modules were retained for downstream analyses to ensure biological relevance. CRISPR spacer match methods were used to link viral operational taxonomic units (vOTUs) and putative host MAGs. CRISPR spacers were identified and recovered from MAGs using CRISPRone [41]. All spacers were matched to vOTUs using BLASTn (v2.16.0) with a strict threshold value set at 97% identity, 90% coverage, and 1 mismatch target sequence, achieving the linkage of viruses to their hosts. The methods for recovering viruses from 828 metagenomes can be found in SI.

### Microbial abundance profiling and correlation network construction

The 136 high-quality MAGs were clustered at a 95% ANI threshold (species-level) using dRep (v3.2.2) with a minimum alignment fraction of 30%. For each cluster, the MAG with the highest quality score (QS = completeness −5 × contamination) was used as the representative for each species. The relative abundance of representative MAGs representing each species was quantified using CoverM (v0.6.1) with parameters -contig, -min-read-percent-identity 0.95, -min-read-aligned-percent 0.75, -contig-end-exclusion 0, and -m relative_abundance. Detection limits were governed by the default settings of CoverM, which require that at least 10% of a reference genome be covered by at least one read in order to report a non-zero abundance. Given that the 828 metagenomic datasets included time-series or replicate samples from the same WWTPs, we applied a filtering strategy to ensure that only one representative sample per WWTP was retained. Specifically, for WWTPs having multiple samples, we identified and retained the sample closest to the within-WWTP centroid based on Bray-Curtis dissimilarity, yielding a final sample set representing 340 distinct WWTPs, which was used for subsequent diversity and biogeographic analyses. For each continent, the proportion of a species/clade was calculated as the summed relative abundance of each species/clade in all WWTP samples in the continent, which was then divided by the total relative abundance of all *Ca*. Accumulibacter species/clade in all WWTP samples in the continent. Bray-Curtis dissimilarity matrices were calculated based on microbial abundance profiles and used to perform principal coordinates analysis (PCoA) with the scikit-bio (skbio) package in Python. Additionally, bacterial community profiles were generated using Sylph (v0.6.1) [42] with default settings on quality-filtered reads and taxonomic assignments based on the GTDB release 214 reference database. Relative abundances were calculated as the proportion of reads assigned to each taxon, and aggregated to the genus level. Pairwise correlations between genera were calculated using Spearman's rank correlation implemented in Python (v3.11, SciPy package v1.11.3, spearman function). Correlations with coefficients |ρ| > 0.3 and false discovery rate (FDR)-adjusted *P* values <0.05 were retained. Genera positively correlated with *Ca*. Accumulibacter were identified based on these filtered correlations.

### Amino acid auxotrophy prediction and carbon source utilization simulations

Draft genome-scale metabolic models (GEMs) were reconstructed for each *Ca*. Accumulibacter MAG using gapseq (v1.4.0) [38] to systematically infer auxotrophy profiles and substrate utilization potentials. Enzymatic functions were predicted by aligning genome-encoded protein sequences to curated reference databases (UniProt, BRENDA, TCDB) using BLAST in gapseq, with default cutoffs of bitscore ≥200 and query coverage ≥75%. Pathway presence was assessed using completeness thresholds applied to MetaCyc, KEGG, and ModelSEED pathway definitions, with a preference for MetaCyc-based pathways due to their high specificity. Transporter functions were similarly inferred based on homology against the TCDB database using the same alignment criteria. The resulting SBML-format GEMs were curated and used as inputs for dynamic cycle-based simulations.

To evaluate amino acid auxotrophy, each genome-scale metabolic model was simulated under aerobic conditions in a defined minimal medium supplemented with acetate and inorganic ions. Initially, uptake of all 20 proteinogenic amino acids was permitted. For each simulation, individual amino acid uptake reactions were sequentially blocked with all other conditions unchanged. A model was classified as auxotrophic for a given amino acid if the predicted growth rate dropped below 0.01 h^−1^; otherwise, it was considered prototrophic.

For substrate utilization potential assessment, we referred to the methodological framework proposed in previous studies [43, 44], implemented a time-resolved simulation framework that mimics a canonical EBPR cycle: a 1.5-hour anaerobic phase followed by a 3.5-hour aerobic phase, with a temporal resolution of 0.2 h per step. Key extracellular (e.g. carbon sources, CO_2_) and storage compounds (e.g. glycogen, polyphosphate, PHAs) and biomass were updated dynamically at each step using forward Euler integration. Other intracellular metabolites remained at quasi-steady state. All simulations were carried out using COBRApy v0.26.3. Anaerobic conditions were simulated by setting the oxygen uptake flux (O₂ exchange) to 0 mmol·gDW^−1^·h^−1^ and all electron transport chain (ETC) reactions to 0. Aerobic conditions were simulated by allowing oxygen uptake while blocking carbon source uptake. A constant non-growth associated maintenance energy demand of 0.398 mmol ATP·gDW^−1^·h^−1^ was applied. During the anaerobic phase, the objective was to minimize the overall fluxes under a fixed substrate-uptake rate to approximate energy-efficient storage metabolism. During the aerobic phase, the objective was to maximize biomass formation using a parsimonious FBA formulation. All simulations were performed under consistent medium conditions to enable cross-strain comparisons. The purpose of these simulations was to evaluate the metabolic feasibility of each substrate under conditions approximating EBPR operation, rather than to reproduce exact kinetics or population-level dynamics. The models and simulation scripts used in this study are available at: https://github.com/Xiaojing-Xie/FBA_PAOS and https://doi.org/10.57760/sciencedb.18043.

### Experimental enrichment and substrate utilization assays

Three sequencing batch reactors (SBRs) were operated to experimentally validate key findings. Activated sludge collected from a full-scale WWTP in Guangzhou was used as an inoculum of SBR1 (in which *Ca*. Accumulibacter nuwaii SCUT-4 was recovered), a 5 L laboratory-scale reactor, which was fed with glucose as a carbon source. Enrichment cultures from SBR1 were sampled on Days 80 and 116, respectively, for anaerobic-aerobic cycle studies, metagenomic sequencing, and metatranscriptomic analyses. SBR2 is a 5.4 L reactor (where *Ca*. Accumulibacter similis SCELSE-1 was recovered) [7], inoculated with an EBPR enrichment culture, and fed with acetate as a carbon source, with amino acid utilization assays performed on Day 149. SBR3 (working volume 4.5 L) (where *Ca*. Accumulibacter cognatus SCUT-2 was recovered) [6] was seeded with activated sludge from the same WWTP in Guangzhou with acetate as a carbon source. Anaerobic-aerobic cycle experiments were conducted to evaluate the ability of *Ca*. Accumulibacter to utilize lactate and succinate. In addition, three additional lab-scale reactors were operated for the enrichment of *Ca*. Accumulibacter (where SCUT-4, SCUT-5, SCUT-6, SCUT-7 and SCUT-8 are recovered in this study). Additionally, two MAGs (SCELSE-11 and SCELSE-14) are newly recovered from previously operated enrichment reactors [45]. In total, seven high-quality MAGs were newly recovered from our lab-scale bioreactors, which are included in the high-quality MAG dataset for *Ca*. Accumulibacter. Detailed reactor operating conditions, anaerobic-aerobic cycle protocols, sample collection procedures, and sequencing methodologies for all these reactors are provided in the SI.

## Results and discussion

### Phylogenomic reconstruction and taxonomic refinement reveal hidden diversity

We reconstructed MAGs from 828 metagenomes from globally distributed WWTPs (Dataset 1 Sheet1), recovering 24 536 MAGs, including 104 high-quality MAGs taxonomically assigned to *Ca*. Accumulibacter. Additionally, 7 high-quality MAGs were newly retrieved from our lab-scale bioreactors (operational parameters are provided in the SI). After dereplication against 94 publicly available high-quality MAGs in the NCBI (assessed in October 2024), we constructed a dataset comprising 136 non-redundant high-quality (completeness >90%, contamination <5%) *Ca*. Accumulibacter MAGs, expanding its genome repertoire by 72%, representing the most comprehensive and globally representative MAG collection for *Ca*. Accumulibacter to date (SI Table. S1).

**Figure 1:**
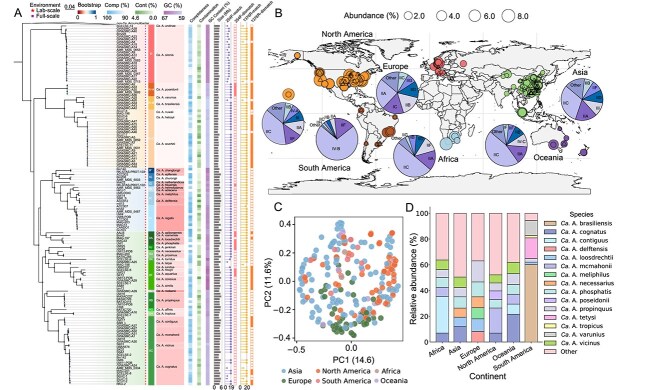
Phylogenomics and global biogeographic distribution of *Ca.* Accumulibacter. (A) A maximum-likelihood phylogenomic tree constructed from concatenated single-copy core genes across 136 *Ca*. Accumulibacter MAGs and reference MAGs. Shapes denote MAG origin (lab-scale vs. full-scale WWTPs). Heatmaps and bar plot indicate genome completeness, contamination, GC content, and primer-template mismatches for the widely used *ppk1* qPCR primers (254F and 1376R). (B) Global biogeographic distribution of *Ca*. Accumulibacter across six continents based on 340 refined WWTPs. Circle size represents relative abundance in metagenomes; hollow triangles represent sites with zero abundance and pie charts indicate clade composition within each continent. (C) PCoA based on Bray-Curtis dissimilarity showing compositional separation of *Ca*. Accumulibacter populations across samples. (D) Proportional distribution of *Ca*. Accumulibacter species across continents.

The phylogenomic tree based on *ppk1* sequences (SI Fig.S1) revealed that 33 MAGs formed a distinct, deeply branching clade, phylogenetically divergent from previously described ones. This clade is designated as Clade IV which is further subdivided into three subgroups (IV-A, IV-B, and IV-C) to capture its internal diversity. The designation is supported by an average *ppk1* nucleotide similarity value of 83.0% to Clades I-III, compared to the average similarity of 84.7% among those existing clades (Clades I-III), with a high average internal similarity (90.1%) sharing among Clade IV members (SI Table S2), consistent with previous criteria for clade-level resolution [3]. We also observed that 22 MAGs clustered within Clade III [18], representing an eight-fold expansion of this lineage, suggesting its substantially underestimated diversity and ecological significance. A majority of MAGs recovered from full-scale WWTPs fell within Clades III and IV (Fig. 1A), highlighting their overlooked roles in engineered wastewater ecosystems. We also reported the recovery of high-quality MAGs (completeness >90% and contamination <5%) assigned to Clades ID and IF, which had previously been known only from *ppk1* clone sequences [12], filling major gaps in the phylogenetic landscape of *Ca*. Accumulibacter. Given this expanded genomic diversity, especially from previously uncharacterized clades (Clades III and IV), we re-evaluated the performance of widely used *ppk1* qPCR primers (254F/1376R) [46]. We found widespread mismatches (SI Fig.S2). Specifically, 30% (41) of the *ppk1* sequences carried ≥1 mismatch at both primer sites, with 26 of these sequences derived from newly recovered MAGs, primarily within Clades III and IV, which are comparable to previously reported primer mismatches for Clade III members [18]. Such mismatches are expected to impair amplification efficiency and contribute to the persistent underrepresentation of these lineages in amplicon-based studies, calling for the development of clade-specific primers to ensure comprehensive and accurate monitoring.

The POCP, although frequently employed with a 50% threshold to delineate genera [47], yielded inconsistent results: some intra-genus comparisons within *Ca*. Accumulibacter fell as low as 47%, whereas inter-genus comparisons (e.g. with *Zoogloea ramigera*) occasionally exceeded the cutoff (SI Fig. S3). In contrast, average amino acid identity (AAI) provided clearer resolution, with all intra-genus comparisons exceeding 72% and comparisons to the nearest outgroups consistently below that threshold (SI Fig. S4). ANI within *Ca*. Accumulibacter MAGs ranged between 77–95%, consistent with previously reported values for this lineage [3, 48], aligning well with genus- and species-level thresholds (SI Fig. S5). The observed incongruence between POCP and ANI/AAI values within *Ca*. Accumulibacter likely stems from the different sensitivities of these metrics to genome size variation and the extent of accessory genes. POCP quantifies the proportion of shared proteins relative to the combined size of two MAGs. As such, it is strongly influenced by disparities in genome sizes, which can lead to underestimated similarity in cases when one MAG is substantially larger than the other. For instance, UW14 has a genome size of 5.35 Mb, whereas that of GWASMC-A46 is only 2.83 Mb, resulting in an extremely low POCP value of 47.6%, although their corresponding ANI and AAI values are 77.5% and 73.3%, respectively. In addition, pangenome analysis revealed that only 1.6% of gene families (1138) are conserved across all *Ca*. Accumulibacter MAGs. 49.2% are unique to single MAGs, reflecting a high extent of accessory gene repertoire. Collectively, these findings highlight the limitations of POCP in resolving taxonomic boundaries in accessory gene-rich taxa and support the adoption of AAI values as a more robust and discriminative genus-level marker under such circumstances.

Applying the standard 95% ANI cutoff [49], we delineated 40 species-level clusters within the genus. Twenty-one of them (56% of MAGs) represent previously undescribed species, most of which were identified for newly recovered MAGs. Even in the absence of complete 16S rRNA gene sequences, congruence between *ppk1* and genome-wide phylogenies enabled robust species delineation, the etymology of species names is provided in SI Table S1. Collectively, these results redefine *Ca*. Accumulibacter's taxonomic landscape, expose longstanding classification biases that underpin future ecological and engineering investigations of this pivotal functional group in EBPR systems.

### Global distribution of *Ca*. Accumulibacter highlights its central role

Due to the limited resolution of 16S rRNA genes in resolving species-level diversity, the intra-lineage level global distribution of *Ca*. Accumulibacter remains incompletely understood. We systematically characterized the global biogeographic patterns of *Ca*. Accumulibacter at a higher phylogenetic resolution (Dataset 1 Sheet2). The strong correlation between species-resolved and genus-level relative abundance estimates (Spearman ρ = 0.95, SI Fig. S6) validated the robustness of the dataset. To avoid potential biases arising from repeated sampling from the same WWTPs, we selected 340 representative samples (each represents one WWTP) from the full set of 828 metagenomes for subsequent analyses. Across WWTPs worldwide, *Ca*. Accumulibacter was consistently identified as a core genus, defined by high relative abundances and widespread occurrences (Fig. 1B). It exhibited considerable ecological prominence, with higher relative abundances than the widely distributed PAO *Ca*. Phosphoribacter in 51.8% of 340 EBPR WWTPs, particularly across continents outside Asia and Europe (SI Figs S7 and S8, SI Tables S3 and S4). These findings underscore the ecological competitiveness of *Ca*. Accumulibacter in diverse treatment environments and its indispensable role in biological phosphorus removal. *β*-diversity and Bray-Curtis dissimilarity revealed different biogeographic structuring (Fig. 1C). European WWTPs displayed low dispersion and high species-level homogeneity, indicating stable and conserved communities. In contrast, Asian WWTPs exhibited exceptional intra-regional diversity, with high *β*-dispersion and a relatively even distribution of multiple *Ca*. Accumulibacter species (Fig. 1D), lacking a dominant taxon.

Despite near-ubiquitous occurrence across global WWTPs, *Ca*. Accumulibacter exhibits marked ecological structuring at finer phylogenetic scales. Clades II and IV consistently dominated across continents. Clade I was rarely detected. Clade III exhibited moderate relative abundances restricted to specific regions (Fig. 1B and SI Fig. S9). This uneven distribution underscores the adaptive advantage of certain lineages under engineered environmental conditions. Among all identified species, *Ca*. Accumulibacter loosdrechtii was the most globally widespread, being detected in 185 WWTPs. *Ca*. Accumulibacter mcmahonii exhibited the highest cumulative relative abundance worldwide. These two dominant species were not captured in previous 16S rRNA gene amplicon sequencing-based surveys, highlighting major gaps in previous surveys and demonstrating that key functional populations in *Ca*. Accumulibacter may have been systematically overlooked. Biogeographic comparisons revealed contrasting distribution patterns among species. Some lineages, including *Ca*. Accumulibacter propinquus, *Ca*. Accumulibacter phosphatis, and *Ca*. Accumulibacter cognatus, were detected across four or more continents (Dataset 1 Sheet3), suggesting that they represent globally conserved core taxa with broad ecological adaptability. In contrast, 11 species exhibited geographically restricted distributions, with no detection in at least half of the continents. For instance, *Ca*. Accumulibacter poseidonii was only detected in North America (Fig. 1D), implying that regional environmental selection plays a pivotal role in shaping *Ca*. Accumulibacter community structure.

To minimize potential confounding introduced by process heterogeneity (EBPR vs. non-EBPR systems), we refined the analysis on a curated subset of 237 WWTPs confirmed as operating EBPR or exhibiting phosphorus removal activities. The EBPR-only dataset recapitulated the major phylogenetic and biogeographic patterns observed globally. All 41 species were detected within this dataset. At the clade level, Clade IIC remained dominant in Africa, Asia, North America, and Oceania (SI Fig. S9). In contrast, Clade IIA was most abundant in Europe. Clade IV-B dominated South America (Dataset 1 Sheet4). At the species level, *Ca*. Accumulibacter cognatus showed the highest cumulative relative abundances in Oceania and Asia. *Ca*. Accumulibacter propinquus and *Ca*. Accumulibacter mcmahonii predominated Europe and North America, respectively. However, given the limited numbers of EBPR plants in Africa (6), South America (7) and Oceania (9), the clade-/species- level predominance patterns on these continents deserve further validation.

Common species such as *Ca*. Accumulibacter loosdrechtii and *Ca*. Accumulibacter phosphatis remained widely distributed, being detected in 135 and 131 EBPR systems, respectively. Eleven *Ca*. Accumulibacter species still exhibited geographically restricted distributions, with cumulative relative abundance of zero in at least three continents, suggesting that regional environmental filtering may contribute to shaping community composition, even under broadly comparable functional conditions. Although these patterns should be interpreted with caution, they nonetheless highlight potential biogeographic influences beyond process-specific effects. This geographic filtering was accompanied by lineage-specific functional adaptations. For instance, low-relative-abundance species such as *Ca*. Accumulibacter heboyii and *Ca*. Accumulibacter jenkinsii [13] were primarily recovered in atypical EBPR configurations (e.g., WWTPs operated with reduced aeration). Their narrow ecological distributions and niche-specific occurrence suggest functional specialization and point to their potential utility as bioindicators of marginal or perturbed system states. The overall consistency between the full dataset and EBPR-filtered analyses indicates that *Ca*. Accumulibacter represents a phylogenetically and ecologically diverse lineage shaped by region-specific selective pressures, highlighting the critical importance of species-level resolution for revealing functional differentiation, tracking process-relevant lineages, and developing ecological diagnostics.

### Divergent evolutionary strategies shape genomes of *Ca*. Accumulibacter

Pangenome analysis of 136 *Ca*. Accumulibacter MAGs revealed a total of 73,022 orthologous gene families, reflecting the remarkably high genetic diversity of the genus (Dataset 2 Sheet1 and Sheet2). Among these, 49.2% of gene families were present in single MAGs (SI Fig. S10), indicating that the pan-genome remains open. In contrast, single-copy orthologs accounted for 99.8% of the pan-genome, underscoring the highly conserved and low-redundancy nature of its genomic architecture. Using a ≥ 80% genome presence threshold (n ≥ 109), we identified 1138 core genes, representing the conserved metabolic backbone of *Ca*. Accumulibacter (SI Fig. S10). These include genes central to phosphate uptake and storage (e.g. *pitA*, *ppk1*, *ppk2*), polyhydroxyalkanoate (PHA) (*phaA*, *phaB*, *phaC*) and glycogen (*glgA*, *glgB*, *glgC*) metabolisms [31, 50]. The ubiquitous conservation of these functions across clades underscored their essential role in conferring the PAO phenotype. However, their uniformity suggests that they are unlikely to be the major drivers of ecological diversification among clades.

**Figure 2:**
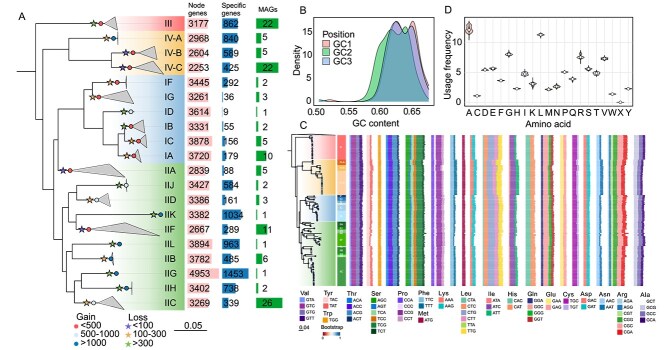
Genomic features and evolutionary patterns of *Ca*. Accumulibacter. (A) A maximum likelihood phylogenetic tree constructed using concatenated single-copy core genes from 136 *Ca*. Accumulibacter MAGs. The tree is annotated with the number of MAGs, clade-specific core genes (specific genes), clade-ancestral genes (node genes), and branch-level gene gain and loss events inferred by count. (B) GC content distributions at the first (GC1), second (GC2), and third (GC3) codon positions across all genomes. (C) Codon usage heatmap across clades. Column represent codons grouped by amino acids. (D) Amino acid usage frequency across *Ca*. Accumulibacter MAGs.

In contrast to the conserved core genome, we observed pronounced lineage-specific patterns of gene acquisition and loss (Fig. 2A). Among lineages with ≥2 MAGs, Clade III was particularly distinctive, harboring the highest number of clade-specific genes (862, Dataset 2 Sheet3), whereas showing no net gene gain at its ancestral node (0 gained, 375 lost), suggesting the specificity primarily reflects long-term retention of ancestral functionalities rather than recent acquisition. In contrast, members of Clade II exhibited dynamic genomic reconfiguration, characterized by substantial gene gain and loss. For example, Clade IIH gained 1,138 genes and retained 738 clade-specific genes, particularly enriched in functions related to inorganic ion transport and DNA repair, traits likely advantageous under oxidative stress or nutrient-limited conditions. Similarly, Clades IIB and IIJ demonstrated major expansions, acquiring 1285 and 937 genes respectively. This dynamic genome evolution may underpin the widespread ecological success of Clade II across WWTPs. Within Clade IV, contrasting trajectories were observed. IV-A experienced extensive gene acquisition (1,056 gained), suggesting niche expansions, whereas IV-B and IV-C followed a more balanced mode with relatively balanced gene gain (349 and 290, respectively) and minimal gene loss (<55 lost). Conversely, Clade I members exhibited minimal lineage-specific divergence, with consistently low numbers of unique genes (e.g. IB: 55; IC: 156; IA: 179) and limited net gene acquisition. These clades appear to have retained ancestral functional frameworks with little evidence of specialization, potentially explaining their narrower global distribution relative to Clade II, III, and IV members.

We examined how genome composition contributes to adaptive potentials. Among the 136 MAGs, the GC content ranged from 59.1% to 66.5%, and genome sizes varied between 2.83 and 5.55 Mb (Fig. 1A), highlighting substantial lineage-specific divergence. Clade IV exhibited significantly reduced genome size (much smaller than the smallest one reported previously, i.e., 3.7 Mb [11]) and lower GC content, particularly in Clade IV-C (e.g. GWASMC-A46, 2.83 Mb, 59.9% GC). Clade IV members, which were primarily recovered from full-scale WWTPs (26/32), were detected in 69 of the 237 EBPR-related WWTPs, suggesting that streamlined genomes and elevated AT content may confer a selective advantage under highly competitive conditions by minimizing replication and repair costs [51]. Moreover, strong tetranucleotide compositional differences were observed across MAGs, with an overrepresentation of CGGC and CGCC motifs and near-complete absence of TTAA and CTAG (SI Fig. S11). This bias may enhance DNA stability and limit exposure to restriction enzyme cleavage, thus promoting resilience under phage predation and environmental stress [52]. GC content across all three codon positions was remarkably consistent (GC1/2/3 of 62.6–62.7%, CV < 3.1%, Fig. 2B), deviating from typical prokaryotic patterns where GC3 tends to show elevated variability [53], and suggesting strong purifying selection to maintain translational stability under fluctuating wastewater treatment conditions.

We detected pervasive codon usage bias across MAGs. For example, the ATC codon, as the optimal codon for isoleucine, was preferentially used (Fig. 2C), likely enhancing translation efficiency under rapid-growth conditions [54]. Distinct codon usage profiles were evident among clades, likely reflecting adaptive strategies to different ecological niches. Clade IV showed preferential use of the glutamine codon CAA (Fig. 2D), a pattern associated with adaptation to low-temperature environments, whereas Clade IIK exhibited an elevated Glu/Asp ratio (>1), a molecular hallmark linked to thermotolerance [55]. Amino acid usage was highly conserved across *Ca*. Accumulibacter species and clades, with alanine and leucine being preferentially utilized, consistent with their low biosynthetic costs (1 and 3 ATP, respectively) [56]. These patterns suggest an integrated genomic strategy that balances metabolic economy and functional stability, enabling efficient resource use and ecological resilience in the WWTPs.

### Auxotrophy and community-level dependency in *Ca*. Accumulibacter

**Figure 3:**
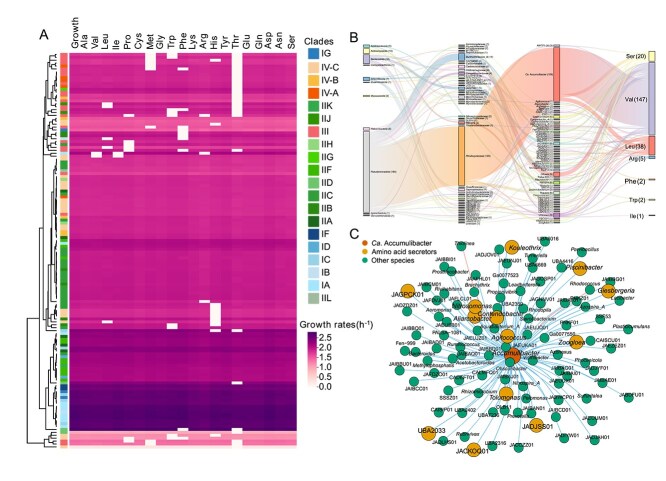
Amino acid auxotrophy and metabolic interactions in *Ca*. Accumulibacter. (A) Predicted amino acid auxotrophy and growth response across *Ca*. Accumulibacter MAGs. The first column (``growth'') represents the predicted specific growth rate when all amino acids are supplemented. Subsequent columns correspond to individual amino acids indicate the predicted specific growth rate (normalized, h^−1^) under conditions lacking the respective amino acid. (B) Sankey diagram showing the taxonomic distribution of all predicted amino acid-secreting microorganisms. Numbers in parentheses represent the number of MAGs assigned to each taxonomic group. (C) Co-occurrence network of *Ca*. Accumulibacter and associated microbial genera across 828 metagenomes. Each node represents a genus, and edges indicate significant co-occurrence correlations (Spearman *P* > 0.3).

*Ca*. Accumulibacter remains refractory to axenic cultivation, limiting empirical characterization of its physiology. To elucidate the underlying ecological and metabolic constraints, we reconstructed 136 GEMs and systematically assessed auxotrophy, and cross-species metabolic complementarity. Approximately 60% of strains were fully prototrophic (Fig. 3A), capable of synthesizing all 20 proteinogenic amino acids under minimal medium conditions, reflecting deep conservation of core biosynthetic pathways that may underpin the lineage's ecological persistence. The removal of threonine (Thr) led to the most pronounced reduction in growth rate, dropping to an average of 1.54 h^−1^ (Fig. 3A), despite the presence of intact biosynthetic pathways. This amino acid occupies central nodes in carbon (e.g. the homoserine-derived Thr branch) metabolism, and its exogenous supply likely alleviates flux bottlenecks at these key regulatory hubs [57]. In contrast, the addition of energetically expensive aromatic amino acids such as tryptophan and phenylalanine provided limited benefit [58], indicating that metabolic reliance is more closely tied to network topology than to biosynthetic cost alone. Across the population, 40% of models exhibited auxotrophy for one or more amino acids (Fig. 3A), most frequently for Thr and histidine (His), with Clades III and IV-C showing particularly high auxotrophy prevalence. Strains originating from full-scale WWTPs exhibited a higher incidence of auxotrophy than those from laboratory systems, suggesting that diverse microbial communities may relieve selection pressure on biosynthetic pathways. This supports a model of "symbiosis-optimized reductive evolution", where gene loss is driven by ecological integration rather than genome streamlining for energy savings.

To investigate the ecological basis of auxotrophy of *Ca*. Accumulibacter, we reconstructed 1872 GEMs, comprising 136 from *Ca*. Accumulibacter and 1736 from co-occurring taxa across 61 WWTPs. Among these, 218 models from 58 genera were predicted to possess amino acid secretion capacity, potentially supporting the growth of *Ca*. Accumulibacter (Fig. 3B). One hundred and ten models belonged to *Ca*. Accumulibacter, suggesting that different lineage members (Dataset 3 Sheet1 and Sheet2), despite exhibiting auxotrophic traits, may also support the growth of each other. Leveraging the global distribution data, we further identified 102 species showing strong positive correlations with *Ca*. Accumulibacter in terms of occurrences and relative abundance (Spearman ρ > 0.3, Fig. 3C and Dataset 3 Sheet3). Among them, 13 species, such as *Ca*. Contendobacter, *Zoogloea*, and *Nitrosomonas*, were predicted to secrete key amino acids including leucine, valine, and serine (Fig. 3B), potentially supporting the proliferation of *Ca*. Accumulibacter. Contrary to the conventional hypothesis, auxotrophic strains of *Ca*. Accumulibacter did not exhibit significantly smaller genomes compared to prototrophs, indicating that the loss of amino acid biosynthetic pathways does not result from genome streamlining driven by energy constraints. Instead, the metabolic evolution of *Ca*. Accumulibacter appears to reflect a form of "symbiosis-optimized reduction", in which genome simplification selectively targets biosynthetic functions that can be externally supplemented within complex microbial communities.

### Broad carbon metabolic potential of *Ca*. Accumulibacter

Accurate characterization of carbon substrate preferences is essential for understanding its ecological niche specialization and designing low-carbon, high-efficiency EBPR systems. Previous modeling studies have successfully simulated co-substrate utilization [43] and temperature effects [44], but are limited in capturing core metabolic pathways and resolving lineage-specific variability, thereby restricting the exploration of non-canonical metabolic traits. To solve this problem, predictions are made based on genome-scale models reconstructed using gapseq, allowing for a systematic and comparative evaluation of substrate utilization potential across different members. To mitigate prediction biases due to MAG incompleteness, we specifically analyzed substrates showing clear patterns of consistent utilization or widespread deficiency across lineages.

Consistent with their classical PAO phenotype [10], all major *Ca*. Accumulibacter lineages showed conserved metabolic potential for short-chain fatty acids, including acetate and propionate. Widespread utilization capabilities for ethanol and malate were also observed. In contrast, glycerol utilization exhibited significant lineage-dependent variability, primarily due to lineage-specific absences of requisite glycerol transporter systems (i.e., the glycerol uptake facilitator protein, *glpF*). Complex substrates such as cellulose and aromatic hydrocarbons (benzoate, phenol, toluene) were beyond the catabolic capabilities of most members. SK01 from Clade IIC uniquely encoded cellulose degradation machinery, exhibiting a biomass yield of 0.152 (gDW/C-mmol), including endoglucanase-like enzymes, enabling cellulose hydrolysis into oligomers subsequently transported via putative cellobiose-specific transporters (i.e., MFS transporter). To the best of our knowledge, this represents the first documentation of cellulose catabolic potential in *Ca*. Accumulibacter members, suggesting evolutionary expansion of substrate range under selective environmental pressures.

**Figure 4:**
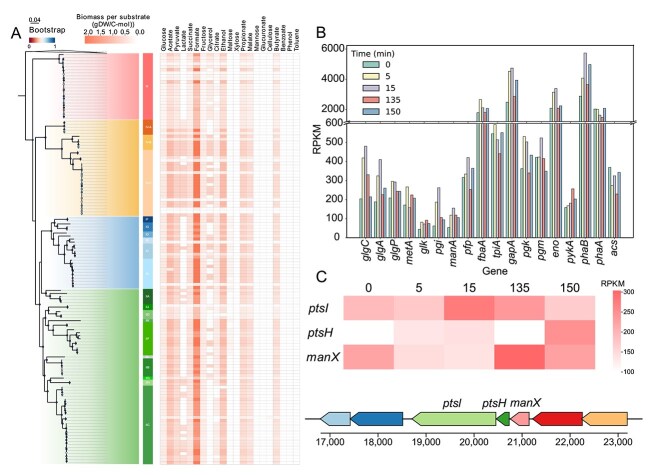
Carbon source utilization characteristics of *Ca*. Accumulibacter. (A) Carbon utilization potential of *Ca*. Accumulibacter. The left panel shows a heatmap of predict biomass yield per substrate across 21 carbon sources based on genome-scale metabolic modeling. (B) Transcriptional levels of genes involved in glycolysis, PHA and glycogen synthesis of *Ca*. Accumulibacter BATAC285 across a full anaerobic-aerobic cycle in glucose-fed bioreactor (*glgC*, glucose-1-phosphate adenylyltransferase gene; *glgA*, glycogen synthase gene; *glgP*, glycogen phosphorylase gene; *metA*, homoserine O-succinyltransferase gene; *glk*, glucokinase gene; *pgi*, glucose-6-phosphate isomerase gene; *manA*, mannose-6-phosphate isomerase gene; *pfp*, pyrophosphate-dependent phosphofructokinase gene; *fbaA*, fructose-bisphosphate aldolase gene; *tpiA*, triosephosphate isomerase gene; *gapA*, glyceraldehyde-3-phosphate dehydrogenase gene; *pgk*, phosphoglycerate kinase gene; *pgm*, phosphoglycerate mutase gene; *eno*, enolase gene; *pykA*, pyruvate kinase gene; *phaB*, acetoacetyl-CoA reductase gene; *phaA*, acetyl-CoA acetyltransferase gene; *acs*, acetyl-CoA synthetase gene). (C) Gene cluster structure and transcript levels of the phosphoenolpyruvate-dependent sugar phosphotransferase system (PTS) in BATAC285, including the mannose-specific IIA component gene (*manX*), the phosphocarrier protein gene (*ptsH*), and the phosphoenolpyruvate-protein phosphotransferase gene (*ptsI*), in representative strains.

Given that anaerobic fermentation of complex organic compounds within WWTPs commonly generates diverse metabolites [4], including lactate and succinate, we evaluated the capacity of *Ca*. Accumulibacter to utilize these prevalent fermentation products as carbon substrates (Fig. 4A). Metabolic modeling predictions demonstrated heterogeneous utilization profiles, nearly all members effectively metabolized succinate, whereas lactate was metabolizable by only approximately one-third (49 out of 136) of members, primarily limited by the apparent absence of lactate-specific transporter genes *lldP* or *lctP*. To verify these model-based predictions, we conducted batch tests (SI Fig. S12) with an enrichment culture in which *Ca*. Accumulibacter cognatus SCUT-2 was predominant (with a relative abundance of 42.8%) with minimal occurrence of other PAOs or GAOs (*Dechloromonas* 0.1%, *Ca*. Competibacter 0.1%, *Tetrasphaera* undetected, Dataset 4 Sheet1). Consistent with simulations, anaerobic uptake of lactate and succinate was confirmed, yielding phosphorus release-to-carbon uptake ratios of 0.28 and 0.36 P mol/C mol, respectively. Previous studies suggest that certain bacteria may employ shared transporters for structurally similar short-chain fatty acids such as acetate and lactate [59], indicating that GEMs may overlook substrate utilization when transporter promiscuity is not captured. During lactate uptake, we observed a pronounced upregulation of the multiple acetate transporter genes (*actP*), reaching a peak transcription level of 567.8 RPKM under anaerobic conditions. In contrast, the only copy of annotated L-lactate permease gene (*lctP*) exhibited consistently low transcriptional levels (<35 RPKM) across all time points (SI Fig. S13), suggesting *actP* may play a role in lactate transport. For confirmation, we compared carbon uptake rates under acetate-only, lactate-only, and mixed-substrate (lactate: acetate = 1:1) conditions. Acetate/lactate alone (40 mg/L) supported carbon uptake of 83.4 C/g SS/h and 6.8 mg C/g SS/h, respectively. The total carbon uptake rate (56.5 mg C/g SS/h) with mixed-substrate was lower than acetate-only condition. High-performance liquid chromatography (HPLC) analysis further showed significantly delayed lactate uptake in the presence of acetate (SI Fig. S13), indicating potential competition between these two substrates. Collectively, these results suggest that acetate and lactate share the same transport mechanism, implying the involvement of *actP* in lactate transport.

Further simulation of amino acid utilization revealed distinct metabolic capacities. SCELSE-9 was predicted to utilize all 20 proteinogenic amino acids for growth. Clades IID and IA exhibited relatively broad amino acid utilization potentials, with each MAG capable of metabolizing an average of 16 amino acids, whereas Clade III members were predicted to utilize only 12 amino acids on average. Transporter loss was particularly common for aromatic amino acids such as phenylalanine, tryptophan, and tyrosine, suggesting constrained uptake of these substrates. To validate model predictions, an enrichment culture of *Ca*. Accumulibacter similis SCELSE-1 was tested for amino acid utilization in batch experiments [7]. In the enrichment culture, SCELSE-1 was the predominant PAO, with a relative abundance of 45% and minor occurrence of known GAOs or other PAOs (Dataset 4 Sheet2), allowing a direct link of amino acid utilization to *Ca*. Accumulibacter. Eleven amino acids were confirmed to support effective phosphorus release, with the highest rates observed for aspartate (21.3 mg P/g MLSS/h) and glutamate (18.5 mg P/g MLSS/h), followed by asparagine (12.9 mg P/g MLSS/h) and glutamine (10.8 mg P/g MLSS/h). These results were largely consistent with model predictions, with the exception of tyrosine and leucine, which were predicted to be non-utilizable due to the absence of the specific transporter and non-anaerobic uptake (SI Fig. S14), respectively. Several amino acids such as histidine and lysine, which did not induce measurable phosphate release, were also consistently predicted to be insufficient to support biomass production. Auxotrophic strains lacking complete biosynthetic pathways were only able to grow when the missing amino acids were externally provided.

The consistency between model predictions and experimental validation bolstered confidence in the viability of our metabolic reconstructions. Nevertheless, some discrepancies were noted. For example, nearly all genome-scale models predicted the ability of *Ca*. Accumulibacter to utilize glucose, yet canonical glucose uptake via the PTS transporter gene *ptsG* is absent in most genomes [60], and glucose utilization was not observed in short-term batch experiments. To resolve this paradox, we enriched PAOs using glucose as the carbon source for 190 days. An enrichment culture was obtained, with the relative abundance of a MAG closely related to *Ca*. Accumulibacter proximus BATAC285 reaching 5.7% (Dataset 4 Sheet3) and the genus-level relative abundance of *Ca*. Accumulibacter of 9.7%. Batch test with glucose as a carbon source showed highly linked P release and glucose uptake of 8.0 mg/L and 20.36 mg TOC/L, respectively (SI Fig. S15). Whereas previous research suggested that, in complex communities, with glucose as a carbon source, fermentative bacteria might convert glucose into short-chain fatty acids such as acetate, propionate and/or lactate [61], which can subsequently be utilized by *Ca*. Accumulibacter for EBPR.

Detailed inspection of the community members was performed with the identification of several potentially fermentative taxa, including *Arachnia* (MAG SBR1_531.17, 8.8%) and *Propionicimonas* (MAG SBR1_531.68, 3.1%). Metatranscriptomic analysis revealed high transcriptions of key genes in the Wood-Werkman pathway in both MAGs (Dataset 4 Sheet4 and Sheet5), implying their involvement in the fermentation of glucose. Concurrently, significant transcription of the acetate permease gene (*actP*) and those for the glycolysis and PHA synthesis (Fig. 4B) were observed for *Ca*. Accumulibacter proximus BATAC285, implying the utilization of the fermentation products of glucose, such as acetate and/or propionate. Genomic analysis suggested that *Ca*. Accumulibacter proximus BATAC285 lacks a glucose-specific phosphoenolpyruvate phosphotransferase system IIBC component gene (*ptsG*), but encodes a substitutive mannose-specific IIA component gene (*manX*) (Fig. 4C), which is located upstream of the phosphocarrier protein gene (*ptsH*) and the phosphoenolpyruvate-protein phosphotransferase gene (*ptsI*).

Previous studies showed that although ManX can mediate glucose transport, the uptake rate is significantly low [62]. Metatranscriptomic data revealed notable transcription of *manX*, *ptsH*, and *ptsI* (Fig. 4C), suggesting that ManX may functionally compensate for PtsG in mediating glucose uptake by *Ca*. Accumulibacter. Whereas, the inefficiency of ManX may have limited *Ca*. Accumulibacter from a competitively direct glucose uptake, particularly with the coexistence of fermentative populations such as *Arachnia* which possess *ptsG*. To assess the broader relevance, we examined the occurrence of *manX* across the *Ca*. Accumulibacter phylogeny. One hundred and thirty-four (out of 136) MAGs encoded *manX* instead of *ptsG*. This may explain why *Ca*. Accumulibacter rarely demonstrated appreciable glucose utilization phenotypes in full- and lab-scale bioreactors.

### Nitrogen metabolism shaped by evolutionary gene loss

**Figure 5:**
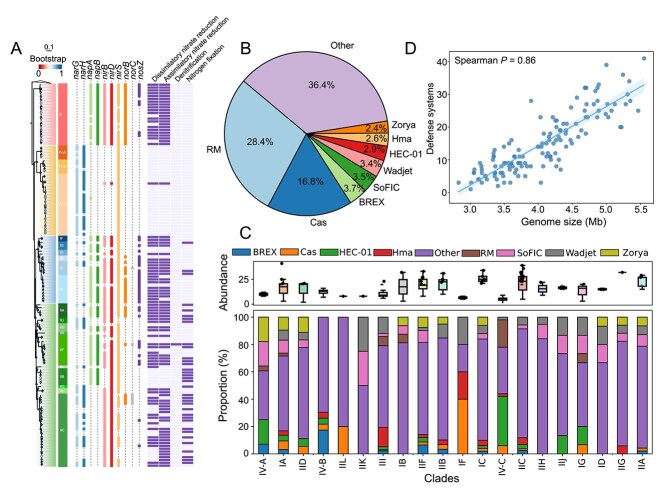
Nitrogen metabolism and antiviral defense systems (DSs) in *Ca*. Accumulibacter. (A) Presence/absence heatmap of genes and modules involved in nitrogen metabolism pathways across 136 *Ca*. Accumulibacter MAGs, including nitrate reduction, denitrification, and nitrogen fixation. (B) Distribution of antiviral defense system categories in *Ca*. Accumulibacter, including restriction-modification (RM) systems, clustered regularly interspaced short palindromic repeats (CRISPR)/CRISPR-associated systems (CRISPR-Cas), abortive infection (Abi), BacteRiophage EXclusion (BREX), and other defense mechanisms. (C) Correlation between genome size and the number of DS modules. (D) Proportional and total DS content across phylogenetic clades; boxplots display interclade variability in DS abundance and composition.

Our analysis revealed that *Ca*. Accumulibacter lacks genes for nitrification or ammonia oxidation, yet displays marked phylogenetic preferences in nitrate reduction, denitrification, and nitrogen fixation (Fig. 5A). Thirty-six MAGs encoded complete nitrogen fixation operons, predominantly distributed across Clade II (e.g. IIA, IIB, IIC, IIF, and IIJ) and two Clade I representatives, suggesting relatively recent acquisitions followed by recurrent losses of this function under nitrogen-rich conditions in WWTPs. Assimilatory and dissimilatory nitrate reduction pathways were encoded in 82 and 73 MAGs, respectively, with 66 MAGs containing both, indicating considerable metabolic flexibility under redox-fluctuating conditions. In contrast, Clade IV genomes generally lacked genes for nitrogen fixation or nitrate reduction, consistent with genome streamlining and energetic pathway contraction in this lineage.

Differences in nitrate and nitrite reduction capacity are widely considered critical to niche specialization of *Ca*. Accumulibacter [3], and remain one of the most debated aspects of its physiology.

Nitrate reduction is mediated by either the periplasmic NapAB system or the membrane-bound NarGHI complex. Nap has higher nitrate affinity and oxygen tolerance, but does not directly contribute to proton gradient generation (thus incapable of supporting growth), offering metabolic flexibility under dynamic conditions (microaerobic and low nitrate concentrations) [63]. Nar is optimized for high nitrate concentrations, relying on active transport (e.g. NarK, which is encoded by nearly all *Ca*. Accumulibacter MAGs), and is activated only under strict anoxic conditions, tightly coupled to proton translocation and ATP synthesis, supporting anaerobiosis [64]. These systems exhibited clear phylogenetic specificities. Among the 111 MAGs encoding nitrate reduction capability, *napAB* dominated Clades I and II. *narGHI* was prevalent in Clades II and IV, suggesting lineage-specific adaptations in electron acceptor usage and energy conservation strategies (Fig. 5A). *Ca*. Accumulibacter proximus MAG-200 uniquely encoded both systems, indicating metabolic complementarity and flexibility in adapting varying nitrate/oxygen conditions.

The downstream steps of denitrification were more sporadically distributed. The nitrite reductase gene *nirS*, which encodes an iron-dependent cytochrome cd₁ enzyme, was identified in 122 MAGs, indicating widespread conservation, except for a few members of Clades IIC, IIJ, and III. This enzyme operates in conjunction with periplasmic electron carriers such as pseudoazurin and is well-suited for microaerobic conditions [65]. In contrast, no MAGs encoded *nirK*, which encodes a copper-dependent nitrite reductase known for high reactivity but strict anaerobiosis [65]. The absence of *nirK* and the predominance of *nirS* suggest an evolutionary trajectory favoring iron-based nitrite reduction, likely shaped by adaptation to high-carbon, low-oxygen, iron-rich environments typical for wastewater treatment ecosystems. The nitric oxide reductase subunit *norB* was detected in 44 MAGs, mainly within Clades III, IIC, and IIA, whereas *norC* was almost entirely missing (n = 6), suggesting a common bottleneck. The final step, mediated by nitrous oxide reductase (encoded by *nosZ*), was present in 40 MAGs.

Only one MAG, *Ca*. Accumulibacter houyii SCELSE-6, encoded a complete denitrification pathway, relying on *napAB* rather than *narGHI*. This implies an inability to couple denitrification to phosphate uptake via respiratory nitrate reduction. Nineteen MAGs, mostly from Clades IIC, IV, and IIK, completely lacked all denitrification-related genes, suggesting their incapability of nitrate or nitrite respiration. Given that nitrate/nitrite respiration competes with polyphosphate-driven VFA uptake under anaerobic conditions [66], the absence of nitrate/nitrite reduction capacity in these clades may alleviate reducing equivalents (NADH/NADPH) competition [67], enabling more efficient carbon uptake during the short anaerobic phases. These members may persist under anoxic conditions, recognizing the anoxic conditions as ``pseudo-aerobic'' which enables sustained carbon uptake in the presence of nitrate and/or nitrite [66, 67]. Although most MAGs lacked complete denitrification pathways, the distribution of individual denitrification genes may enable community-level cooperation in nitrate reduction. Phylogenetic reconstructions revealed that *napAB* was vertically inherited from the *Rhodocyclaceae* ancestor, whereas *narGHI* and *nirS* were acquired via horizontal gene transfer at the LCA of *Ca*. Accumulibacter. Despite the near-universal presence of *narK*, phylogenetic reconstructions indicate heterogeneous acquisition histories (SI Fig. S16). Two primary patterns were observed: (1) ancestral acquisition followed by clade-specific gene loss (which accounted for the majority of observed cases), and (2) more recent lineage-specific or isolated horizontal gene acquisitions. These patterns indicated that widespread absence of denitrification capabilities in *Ca*. Accumulibacter is more likely attributable to gene loss during evolution, rather than to the lack of horizontal gene acquisition in more recent evolutionary history.

Although *napAB* and *narGHI* exhibited lineage-level preferences, no correlation was observed between *ppk1*-based phylogeny and the denitrification capability, with no stable genotype-to-phenotype associations at the species level. These findings highlight that although nitrogen metabolic potential in *Ca*. Accumulibacter is phylogenetically structured, the integration of these pathways remains highly plastic, complicating efforts to categorize denitrification traits based solely on phylogenetic markers.

### Extensive and diverse antiviral defense repertoires in *Ca*. Accumulibacter

Antiviral DSs serve as key barriers against phage predation [68], underpinning microbial genome stability and persistence in virus-rich WWTPs. We identified 2028 complete DSs across 136 MAGs (Dataset 5 Sheet1 and Sheet2), with an average of 14.9 per MAG and a maximum of 41, substantially exceeding the average (4.31) and maximum (25) observed in other WWTP-associated microbes [69]. These systems encompassed 102 types and 148 subtypes, accounting for 91.9% and 82.2% of all known DS types and subtypes detected in WWTP microbial communities, respectively. This extraordinary enrichment suggests that *Ca*. Accumulibacter has faced intensive phage predation and selection pressures thus has undergone great diversification of antiviral defenses. Restriction-modification (RM) systems (28.4%) and CRISPR-Cas systems (16.8%) were the most prevalent (Fig. 5B), representing enzymatic and adaptive immune strategies that collectively form the core antiviral machinery. Other DS types occurred at lower frequencies (<5%) and often exhibited restricted distributions linked to specific lineages or environmental contexts. For example, the SoFIC system, a cAMP-regulated defense module involved in phage-induced host stress signaling, was universally present in Clades IA and IV-A, yet entirely absent in Clades IIB, IV-B, and IV-C. Despite their broad prevalence, the total number and composition of DSs varied markedly across clades and species (Fig. 5C). Clade IC, for example, encoded significantly more DSs (mean 26.2, SD 5.07) than Clades IF (6.5) or IV-C (5.4), yet considerable heterogeneity was observed even among closely related MAGs, underscoring the dynamic and rapidly evolving nature of these systems. MAGs recovered from laboratory reactors encoded a greater number of DSs on average (17.2) than those from full-scale WWTPs (13.1), suggesting that controlled enrichment conditions may impose stronger or more selective viral pressures. Additionally, DS abundance exhibited a strong positive correlation with genome size (Spearman's ρ = 0.86, *P* < 0.001, Fig. 5D), challenging the prevailing view that genome streamlining in WWTP microorganisms is primarily driven by loss of metabolic functions. Instead, our findings highlight antiviral DSs as a key contributor to genome expansion and complexity, revealing a previously underappreciated impact of virus-host interactions on microbial genome evolution.

To further elucidate the ecological significance of DS diversity, we reconstructed a virus-host interaction network between 149 760 WWTP-derived viruses and 136 *Ca*. Accumulibacter MAGs based on CRISPR spacer matches, providing a valuable resource for understanding phage-host ecology and microbiome engineering. A total of 455 vOTUs were linked to 92 *Ca*. Accumulibacter MAGs, resulting in a host infection rate of 67% (Dataset 5 Sheet3). Although most vOTUs exhibited narrow host ranges, 136 vOTUs were capable of infecting multiple hosts, and 22 were associated with five or more strains, suggesting the presence of broad-host-range phages within this lineage. The virus-host association network was highly uneven: no viral match was detected in Clades ID, IB, IIL, and IIK, whereas certain MAGs displayed disproportionately high viral associations. For instance, BAT3C720 was targeted by 93 vOTUs, followed by UW1 (44), SCUT-4 (28), SCUT-2 (25), and BAT3C415 (23), highlighting strain-specific hotspots of phage predation. To evaluate the functional consequences of these interactions, we analyzed long-term reactor performance [45]. Bioreactors dominated by SCELSE-6, a strain encoding a complete CRISPR system and comprising 25.5% relative abundance, maintained stable phosphorus removal throughout the operational period. Conversely, the system enriched in SCELSE-14, lacking detectable CRISPR arrays, experienced catastrophic failure. These observations suggest increased vulnerability to phage-mediated collapse in defense-deficient consortia. Collectively, our findings underscore the pivotal role of antiviral DSs not only in shaping genome architecture but also in conferring ecological stability and engineering resilience.

## Supplementary Material

SI_for_ISME_wraf278

Dataset_1_wraf278

Dataset_2_wraf278

Dataset_3_wraf278

Dataset_4_wraf278

Dataset_5_wraf278

## Data Availability

The metagenomic datasets generated from WWTPs in this study have been deposited in NCBI under the BioProject No. PRJNA1204190. All the GWASMC MAGs, the genome-scale metabolic models of *Ca*. Accumulibacter, and their predicted MAG-encoded genes have been archived in the Science Data Bank and are accessible at https://doi.org/10.57760/sciencedb.18043. The scripts, and example output files for the dynamic simulation are available at https://github.com/Xiaojing-Xie/FBA_PAOS).

## References

[1] Walton CR, Ewens S, Coates JD et al. Phosphorus availability on the early earth and the impacts of life. *Nat Geosci* 2023;16:399–409. 10.1038/s41561-023-01167-6

[2] Zhang C, Guisasola A, Baeza JA. A review on the integration of mainstream P-recovery strategies with enhanced biological phosphorus removal. *Water Res* 2022;212:118102. 10.1016/j.watres.2022.118102 35091221

[3] Petriglieri F, Singleton CM, Kondrotaite Z et al. Reevaluation of the phylogenetic diversity and global distribution of the genus *Candidatus* Accumulibacter. *mSystems* 2022;7:e00016–22. 10.1128/msystems.00016-22 35467400 PMC9238405

[4] Ruiz-Haddad L, Ali M, Pronk M et al. Demystifying polyphosphate-accumulating organisms relevant to wastewater treatment: a review of their phylogeny, metabolism, and detection. *Environ Sci Ecotechnol* 2024;21:100387. 10.1016/j.ese.2024.100387 38322240 PMC10845257

[5] Wu L, Ning D, Zhang B et al. Global diversity and biogeography of bacterial communities in wastewater treatment plants. *Nat Microbiol* 2019;4:1183–95. 10.1038/s41564-019-0426-5 31086312

[6] Chen L, Wei G, Zhang Y et al. *Candidatus* Accumulibacter use fermentation products for enhanced biological phosphorus removal. *Water Res* 2023;246:120713. 10.1016/j.watres.2023.120713 37839225

[7] Qiu G, Liu X, Saw NMMT et al. Metabolic traits of *Candidatus* Accumulibacter clade iif strain SCELSE-1 using amino acids as carbon sources for enhanced biological phosphorus removal. *Environ Sci Technol* 2020;54:2448–58. 10.1021/acs.est.9b02901 31790213

[8] Ziliani A, Bovio-Winkler P, Cabezas A et al. Putative metabolism of *Ca*. Accumulibacter via the utilization of glucose. *Water Res* 2023;229:119446. 10.1016/j.watres.2022.119446 36516560

[9] Oehmen A, Lemos PC, Carvalho G et al. Advances in enhanced biological phosphorus removal: from micro to macro scale. *Water Res* 2007;41:2271–300. 10.1016/j.watres.2007.02.030 17434562

[10] Chen L, Chen H, Hu Z et al. Carbon uptake bioenergetics of PAOs and GAOs in full-scale enhanced biological phosphorus removal systems. *Water Res* 2022;216:118258. 10.1016/j.watres.2022.118258 35320769

[11] Xie X, Deng X, Chen J et al. Two new clades recovered at high temperatures provide novel phylogenetic and genomic insights into *Candidatus* Accumulibacter. *ISME Commun* 2024;4:ycae049. 10.1093/ismeco/ycae049 38808122 PMC11131965

[12] Mao Y, Graham DW, Tamaki H et al. Dominant and novel clades of *Candidatus* Accumulibacter phosphatis in 18 globally distributed full-scale wastewater treatment plants. *Sci Rep* 2015;5:11857. 10.1038/srep11857 26138542 PMC4490554

[13] Stewart RD, Myers KS, Amstadt C et al. Refinement of the *Candidatus* Accumulibacter genus based on metagenomic analysis of biological nutrient removal (BNR) pilot-scale plants operated with reduced aeration. *mSystems* 2024;9:e01188–23. 10.1128/msystems.01188-23 38415636 PMC10949500

[14] Welles L, Lopez-Vazquez CM, Hooijmans CM et al. Prevalence of `*Candidatus* Accumulibacter phosphatis' type II under phosphate limiting conditions. *AMB Express* 2016;6:44. 10.1186/s13568-016-0214-z 27376945 PMC4932009

[15] Lanham AB, Moita R, Lemos PC et al. Long-term operation of a reactor enriched in accumulibacter clade I DPAOs: performance with nitrate, nitrite and oxygen. *Water Sci Technol* 2011;63:352–9. 10.2166/wst.2011.063 21252442

[16] Camejo PY, Oyserman BO, McMahon KD et al. Integrated omic analyses provide evidence that a ``*Candidatus* Accumulibacter phosphatis'' strain performs denitrification under microaerobic conditions. *mSystems* 2019;4:e00193–18. 10.1128/mSystems.00193-18 30944872 PMC6446978

[17] Yuan J, Deng X, Xie X et al. Blind spots of universal primers and specific fish probes for functional microbe and community characterization in EBPR systems. *ISME Commun* 2024;4:ycae011. 10.1093/ismeco/ycae011 38524765 PMC10958769

[18] Wang Z, Song W, Zhang X et al. Expanding the diversity of Accumulibacter with a novel type and deciphering the transcriptional and morphological features among co-occurring strains. *Appl Environ Microbiol* 2023;89:e0077123. 10.1128/aem.00771-23 37466435 PMC10467341

[19] Dueholm MKD, Nierychlo M, Andersen KS et al. Midas 4: a global catalogue of full-length 16S rRNA gene sequences and taxonomy for studies of bacterial communities in wastewater treatment plants. *Nat Commun* 2022;13:1908. 10.1038/s41467-022-29438-7 35393411 PMC8989995

[20] Chen S, Zhou Y, Chen Y et al. Fastp: an ultra-fast all-in-one fastq preprocessor. *Bioinformatics* 2018;34:i884–90. 10.1093/bioinformatics/bty560 30423086 PMC6129281

[21] Nurk S, Meleshko D, Korobeynikov A et al. Metaspades: a new versatile metagenomic assembler. *Genome Res* 2017;27:824–34. 10.1101/gr.213959.116 28298430 PMC5411777

[22] Langmead B, Salzberg SL. Fast gapped-read alignment with bowtie 2. *Nat Methods* 2012;9:357–9. 10.1038/nmeth.1923 22388286 PMC3322381

[23] Li H, Handsaker B, Wysoker A et al. The sequence alignment/map format and samtools. *Bioinformatics* 2009;25:2078–9. 10.1093/bioinformatics/btp352 19505943 PMC2723002

[24] Kang DD, Li F, Kirton E et al. Metabat 2: an adaptive binning algorithm for robust and efficient genome reconstruction from metagenome assemblies. *PeerJ* 2019;7:e7359. 10.7717/peerj.7359 31388474 PMC6662567

[25] Parks DH, Imelfort M, Skennerton CT et al. Checkm: assessing the quality of microbial genomes recovered from isolates, single cells, and metagenomes. *Genome Res* 2015;25:1043–55. 10.1101/gr.186072.114 25977477 PMC4484387

[26] Chaumeil PA, Mussig AJ, Hugenholtz P et al. Gtdb-tk: a toolkit to classify genomes with the genome taxonomy database. *Bioinformatics* 2019;36:1925–7. 10.1093/bioinformatics/btz848 31730192 PMC7703759

[27] Olm MR, Brown CT, Brooks B et al. Drep: a tool for fast and accurate genomic comparisons that enables improved genome recovery from metagenomes through de-replication. *ISME J* 2017;11:2864–8. 10.1038/ismej.2017.126 28742071 PMC5702732

[28] Prokka ST . Rapid prokaryotic genome annotation. *Bioinformatics* 2014;30:2068–9. 10.1093/bioinformatics/btu153 24642063

[29] He S, Gall DL, McMahon KD. ``*Candidatus* Accumulibacter'' population structure in enhanced biological phosphorus removal sludges as revealed by polyphosphate kinase genes. *Appl Environ Microbiol* 2007;73:5865–74. 10.1128/AEM.01207-07 17675445 PMC2074919

[30] Tonkin-Hill G, MacAlasdair N, Ruis C et al. Producing polished prokaryotic pangenomes with the panaroo pipeline. *Genome Biol* 2020;21:180. 10.1186/s13059-020-02090-4 32698896 PMC7376924

[31] Xie X, Deng X, Chen L et al. Integrated genomics provides insights into the evolution of the polyphosphate accumulation trait of *Ca*. *Accumulibacter Environ Sci Ecotechnol* 2023;20:100353. 10.1016/j.ese.2023.100353 39221073 PMC11361876

[32] Hellewell J, Horsfield ST, von Wachsmann J et al. Celebrimbor: Core and accessory genes from metagenomes. *Bioinformatics* 2024;40:btae542. 10.1093/bioinformatics/btae542 39298479 PMC11422503

[33] Katoh K, Standley DM. Mafft multiple sequence alignment software version 7: improvements in performance and usability. *Mol Biol Evol* 2013;30:772–80. 10.1093/molbev/mst010 23329690 PMC3603318

[34] Minh BQ, Schmidt HA, Chernomor O et al. Iq-tree 2: new models and efficient methods for phylogenetic inference in the genomic era. *Mol Biol Evol* 2020;37:1530–4. 10.1093/molbev/msaa015 32011700 PMC7182206

[35] Csűös M . Count: evolutionary analysis of phylogenetic profiles with parsimony and likelihood. *Bioinformatics* 2010;26:1910–2. 10.1093/bioinformatics/btq315 20551134

[36] Jain C, Rodriguez-R LM, Phillippy AM et al. High throughput ani analysis of 90k prokaryotic genomes reveals clear species boundaries. *Nat Commun* 2018;9:5114. 10.1038/s41467-018-07641-9 30504855 PMC6269478

[37] Pocp-nf HM . An automatic nextflow pipeline for calculating the percentage of conserved proteins in bacterial taxonomy. *Bioinformatics* 2024;40:btae175. 10.1093/bioinformatics/btae175 38561180 PMC11256958

[38] Zimmermann J, Kaleta C, Waschina S. Gapseq: informed prediction of bacterial metabolic pathways and reconstruction of accurate metabolic models. *Genome Biol* 2021;22:81. 10.1186/s13059-021-02295-1 33691770 PMC7949252

[39] Zhou Z, Tran PQ, Breister AM et al. Metabolic: high-throughput profiling of microbial genomes for functional traits, metabolism, biogeochemistry, and community-scale functional networks. *Microbiome* 2022;10:33. 10.1186/s40168-021-01213-8 35172890 PMC8851854

[40] Tesson F, Hervé A, Mordret E et al. Systematic and quantitative view of the antiviral arsenal of prokaryotes. *Nat Commun* 2022;13:2561. 10.1038/s41467-022-30269-9 35538097 PMC9090908

[41] Zhang Q, Ye Y. Not all predicted CRISPR–Cas systems are equal: isolated cas genes and classes of CRISPR like elements. *BMC Bioinformatics* 2017;18:92. 10.1186/s12859-017-1512-4 28166719 PMC5294841

[42] Shaw J, Yu YW. Rapid species-level metagenome profiling and containment estimation with sylph. *Nat Biotechnol* 2024;43:1348–59. 10.1038/s41587-024-02412-y 39379646 PMC12339375

[43] Páez-Watson T, Jansens C, van Loosdrecht MCM et al. Co-substrate utilisation in ``*Candidatus* Accumulibacter'' enhances metabolic fitness in dynamic environments. *Water Res* 2025;287:124401. 10.1016/j.watres.2025.124401 40834730

[44] Páez-Watson T, van Loosdrecht MCM, Wahl SA. Predicting the impact of temperature on metabolic fluxes using resource allocation modelling: application to polyphosphate accumulating organisms. *Water Res* 2023;228:119365. 10.1016/j.watres.2022.119365 36413834

[45] Qiu G, Law Y, Zuniga-Montanez R et al. Global warming readiness: feasibility of enhanced biological phosphorus removal at 35°C. *Water Res* 2022;216:118301. 10.1016/j.watres.2022.118301 35364353

[46] McMahon KD, Yilmaz S, He S et al. Polyphosphate kinase genes from full-scale activated sludge plants. *Appl Microbiol Biotechnol* 2007;77:167–73. 10.1007/s00253-007-1122-6 17671784

[47] Qin QL, Xie BB, Zhang XY et al. A proposed genus boundary for the prokaryotes based on genomic insights. *J Bacteriol* 2014;196:2210–5. 10.1128/jb.01688-14 24706738 PMC4054180

[48] McDaniel EA, Moya-Flores F, Keene Beach N et al. Metabolic differentiation of co-occurring accumulibacter clades revealed through genome-resolved metatranscriptomics. *mSystems* 2021;6:e0047421. 10.1128/mSystems.00474-21 34227830 PMC8407102

[49] Konstantinidis KT, Tiedje JM. Genomic insights that advance the species definition for prokaryotes. *Proc Natl Acad Sci USA* 2005;102:2567–72. 10.1073/pnas.0409727102 15701695 PMC549018

[50] Oyserman BO, Moya F, Lawson CE et al. Ancestral genome reconstruction identifies the evolutionary basis for trait acquisition in polyphosphate accumulating bacteria. *ISME J* 2016;10:2931–45. 10.1038/ismej.2016.67 27128993 PMC5148189

[51] Dietel AK, Merker H, Kaltenpoth M et al. Selective advantages favour high genomic at-contents in intracellular elements. *PLoS Genet* 2019;15:e1007778. 10.1371/journal.pgen.1007778 31034469 PMC6519830

[52] Pride DT, Meinersmann RJ, Wassenaar TM et al. Evolutionary implications of microbial genome tetranucleotide frequency biases. *Genome Res* 2003;13:145–58. 10.1101/gr.335003 12566393 PMC420360

[53] Lassalle F, Périan S, Bataillon T et al. Gc-content evolution in bacterial genomes: the biased gene conversion hypothesis expands. *PLoS Genet* 2015;11:e1004941. 10.1371/journal.pgen.1004941 25659072 PMC4450053

[54] Plotkin JB, Kudla G. Synonymous but not the same: the causes and consequences of codon bias. *Nat Rev Genet* 2011;12:32–42. 10.1038/nrg2899 21102527 PMC3074964

[55] Shen L, Liu Y, Chen L et al. Genomic basis of environmental adaptation in the widespread poly-extremophilic exiguobacterium group. *ISME J* 2024;18:1–19. 10.1093/ismejo/wrad020 PMC10837837 38365240

[56] Arora P, Mukhopadhyay CS, Kaur S. Comparative genome wise analysis of codon usage of *Staphylococcus* genus. *Curr Genet* 2024;70:10. 10.1007/s00294-024-01297-3 39083100

[57] Newsholme P, Procopio J, Lima MM et al. Glutamine and glutamate—their central role in cell metabolism and function. *Cell Biochem Funct* 2003;21:1–9. 10.1002/cbf.1003 12579515

[58] Akashi H, Gojobori T. Metabolic efficiency and amino acid composition in the proteomes of *Escherichia* coli and *Bacillus* subtilis. *Proc Natl Acad Sci USA* 2002;99:3695–700. 11904428 10.1073/pnas.062526999 PMC122586

[59] Leão C, van Uden N. Transport of lactate and other short-chain monocarboxylates in the yeast *Candida* utilis. *Appl Microbiol Biotechnol* 1986;23:389–93. 10.1007/BF00257039 PMC203697 3034152

[60] Xie X, Deng X, Chen L et al. From gene to structure: Unraveling genomic dark matter in *Ca*. *Accumulibacter Environ Sci Technol* 2025;59:628–39. 10.1021/acs.est.4c09948 39699575

[61] Jeon CO, Park JM. Enhanced biological phosphorus removal in a sequencing batch reactor supplied with glucose as a sole carbon source. *Water Res* 2000;34:2160–70. 10.1016/S0043-1354(99)00383-8

[62] Steinsiek S, Bettenbrock K. Glucose transport in *Escherichia* coli mutant strains with defects in sugar transport systems. *J Bacteriol* 2012;194:5897–908. 10.1128/jb.01502-12 22923596 PMC3486086

[63] Stewart V, Lu Y, Darwin AJ. Periplasmic nitrate reductase (napabc enzyme) supports anaerobic respiration by *Escherichia* coli K-12. *J Bacteriol* 2002;184:1314–23. 10.1128/jb.184.5.1314-1323.2002 11844760 PMC134854

[64] Richardson DJ, van Spanning RJM. Ferguson SJ. The prokaryotic nitrate reductases. In Bothe H, Ferguson SJ, Newton We (eds.), *Biology of the Nitrogen Cycle.* Amsterdam: Elsevier, 2007;21–35. 10.1016/B978-044452857-5.50003-5

[65] Sun H, Jiang S. A review on nirs-type and nirk-type denitrifiers via a scientometric approach coupled with case studies. *Environ Sci Processes Impacts* 2022;24:221–32. 10.1039/D1EM00518A 35072673

[66] Carvalho G, Lemos PC, Oehmen A et al. Denitrifying phosphorus removal: linking the process performance with the microbial community structure. *Water Res* 2007;41:4383–96. 10.1016/j.watres.2007.06.065 17669460

[67] Cokro AA, Law Y, Williams RBH et al. Non-denitrifying polyphosphate accumulating organisms obviate requirement for anaerobic condition. *Water Res* 2017;111:393–403. 10.1016/j.watres.2017.01.006 28110143

[68] Bernheim A, Sorek R. The pan-immune system of bacteria: antiviral defence as a community resource. *Nat Rev Microbiol* 2020;18:113–9. 10.1038/s41579-019-0278-2 31695182

[69] Zhang Q, Li J, Tuo J et al. Long-term metagenomic insights into the roles of antiviral defense systems in stabilizing activated sludge bacterial communities. *ISME J* 2025;19:wraf051. 10.1093/ismejo/wraf051 40096540 PMC11980602

