# Supplementary material for: Metagenomic characterization of the metabolism, evolution, and global distribution of Candidatus Accumulibacter members in wastewater treatment plants
Source: ISME J. 2025 Dec 23;19(1):wraf278. doi: 10.1093/ismejo/wraf278 (PMC12766714; doi:10.1093/ismejo/wraf278)
Supplement: SI_for_ISME_wraf278 [file si_for_isme_wraf278.pdf]

Supplementary Information for

Metagenomic characterization of the metabolism, evolution, and global distribution of *Candidatus* Accumulibacter members in wastewater treatment plants

Authors

Xiaojing Xie[1], Liping Chen[1], Jing Yuan[1], Haixin Zheng[1], Lanying Zhang[1], Xiaokai Yu [1], Xianghui Liu [2], Chaohai Wei[1,4], Guanglei Qiu[1,2,3,4,5*]

Affiliations

[1] School of Environment and Energy, South China University of Technology, Guangzhou 510006, China.

[2] Singapore Centre for Environmental Life Sciences Engineering, Nanyang Technological University, Singapore 637551, Singapore.

[3] Guangdong Provincial Key Laboratory of Solid Wastes Pollution Control and Recycling, Guangzhou 510006, China

[4] The Key Lab of Pollution Control and Ecosystem Restoration in Industry Clusters, Ministry of Education, Guangzhou 510006, China

[5] National Joint Research Center for Ecological Conservation and High Quality Development of the Yellow River Basin, Beijing 100018, China

[*]**Corresponding Author:**

qiugl@scut.edu.cn (G.Q.). 382 Waihuan East Road, Panyu District, Guangzhou City, Guangdong Province, China 511436.

This PDF file includes:

Supplementary Text

Tab. S1-S2

Figs. S1 to S15

Legends for Datasets S1 to S5

References

Other Supplementary Materials for this manuscript include the following:

Dataset S1 to S5

Supplementary Text

S1. Creation of the activated sludge metagenomic dataset of global WWTPs

S1.1 Sampling, DNA extract and sequencing of activated sludge from WWTPs. Eighty-one activated sludge samples were collected from municipal wastewater treatment plants (WWTPs) across 40 cities from 31 provinces in China for metagenomic sequencing (Dataset 1). Genomic DNA was extracted using the Fast DNATM 2 mL SPIN Kit for Soil Samples (MP Biomedicals, CA, USA) following the manufacturer's instructions, and stored at -80 °C prior to metagenomic analysis. Sequencing library preparation was performed using a modified version of the Illumina TruSeq DNA Sample Preparation Protocol: 1 μg DNA was sheared on a Covaris S220 to approximately 300 bp, following the manufacturer's recommendation. Size selection was performed on a Sage Science Pippin Prep instrument, using a 2% EtBr agarose cassette and selecting for a tight peak around 400 bp. Each library was tagged with a TruSeq LT DNA barcode (Illumina, CA, USA) to allow for library pooling prior to sequencing. Library quantitation was determined using the Picogreen assay (Invitrogen, CA, USA). The average library size was determined by running the libraries on a Bioanalyzer DNA 7500 chip (Agilent, CA, USA). Library concentrations were normalized to 4 nM and validated by qPCR on a ViiA-7 real-time thermocycler (Applied Biosystems, CA, US), using qPCR primers recommended by Illumina in their qPCR protocol with the Illumina PhiX control library as a standard. Libraries were then pooled and sequenced across two lanes of an Illumina HiSeq2500 sequencing run at a read-length of 250 bp paired-end. Raw reads were submitted to National Center for Biotechnology Information (NCBI) under the BioProject No. PRJNA1204190.

S1.2 Metagenomic data retrieval from NCBI. Additionally, 747 metagenomes of activated sludge from global WWTP were retrieved from the NCBI Sequence Read Archive (https://ncbi.nlm.nih.gov/sra/) (Dataset 1). All the raw metagenomic sequences were processed for quality check and filtration to acquire clean reads using fastp [1]. WWTPs were selected based on the following criteria to minimize possible bias: (1) metagenome samples were not cultured and without additional experiment processes, such as carbon source addition and warming after sampling; (2) Only the control group was considered and the experimental group was excluded; (3) Only paired-end sequencing reads generated by Illumina shotgun platforms with FASTQ format were involved; (4) Accurate coordinate information and habitats were available for spatial analyses; (5) The average read length of metagenomes exceeded 100 base pairs and the total sequence length of metagenome dataset was not less than 100M. These quality control procedures were aimed to minimize possible uncertainty from sampling locations, experimental processes, unexpected contaminations, sequencing methods. A final set of 828 metagenomic sequencing datasets (Dataset 1) were constructed for further analysis.

S2. Lab-scale reactor operation

S2.1 Sequencing batch reactor operation (SBR1). A 5.0 L lab-scale sequencing batch reactors (SBR1) were inoculated with activated sludge from a WWTP in Guangzhou, China for the enrichment of *Ca.* Accumulibacter-related polyphosphate-accumulating organisms (PAOs) with glucose as a carbon source. A slow feed strategy was employed, operating in an 8 h cycle, including a feeding phase (60 min), an anaerobic phase (60 min), an aerobic phase (180 min), and a settling/decant phase (180 min). The influent is a synthetic medium containing an acetate–glucose mixture (1:1, 30 mg TOC /L) as

the carbon source from Day 1 to Day 65. From Day 66 to Day 70, the medium contained only glucose as the sole carbon source (30 mg TOC /L). From Day 71 to Day 149, the glucose concentration was increased to 45 mg TOC /L. In each cycle, two solutions, A and B, were introduced to both reactors at 2000 and 500 ml/cycle, respectively. Solution A is comprised of 4.3L of MilliQ™ water, 200 ml of carbon source solution (as mentioned above) and 500 ml of 10x nutrient stock solution (5L of MilliQ™ water, 10.2 g/L NH_4Cl, 12 g/L $MgSO_4\cdot 7\ H_2O$, 0.1 g/L peptone, 0.1 g/L yeast extract). Solution B is comprised of 25 L of MilliQ™ water, 13.75 ml of 200x phosphate stock solution (66 g/L K_2HPO_4, 51.5 g/L KH_2PO_4), 2 ml of trace solution I (15 g/L of $FeCl_3\cdot 6\ H_2O$, 0.3 g/L of $CuSO_4\cdot 5H_2O$, 0.6 g/L of $MnCl_2$, 1.2 g/L of $ZnSO_4$ and 1.5g/L of $CoCl_2$, acidified with 5ml of concentrated hydrochloric acid) and 1 ml of trace solution II (1.5 g/L of H_3BO_3, 1.8 g/L of KI and 1.2 g/L of Na_2MoO_4). The hydraulic retention time (HRT) and sludge retention time (SRT) were 12 h and 20 d, respectively. The pH was automatically controlled at 7.2±0.3 with an M200 transmitter (Mettler-Toledo, Switzerland) connected to an acid/base (0.5 M HCl/NaOH) dosing system. The dissolved oxygen (DO) values were maintained at 0.8-1.2 mg/L during the aerobic phase by using the same transmitter connected to an air pump. Temperature was controlled at 25°C using a thermostatic water bath.

S2.2 Sequencing batch reactor operation (SBR2) [2]**.** The SBR2 with a working volume of 5.4 L was inoculated with sludge from an EBPR enrichment reactor for the enrichment of *Ca.* Accumulibacter for amino acid utilization capacity tests. A slow feeding strategy was applied for the reactor operation, which has been shown to benefit the proliferation of *Ca*. Accumulibacter. The SBR was operated with 6-h cycles, including a 60-min feed, a 20-min anaerobic, a 180-min aerobic, and a 100-min settling/decant stages. In each cycle, 2.35 L of synthetic wastewater composed of 0.53 L of solution A (containing 1.02 g/L NH_4Cl, 1.2 g/L $MgSO_4\cdot 7\ H_2O$, 0.01g/L peptone, 0.01g/L yeast extract and 6.8 g/L sodium acetate) and 1.82 L of solution B (0. 312 g/L K_2HPO_4, 3 H_2O, 0.185 g/L KH_2PO_4, 0.75 mg/L $FeCl_3\cdot$ 6 H_2O, 0.015 mg/L $CuSO_4\cdot 5H20$, 0.03 mg/L $MnCl_2$, 0.06 mg/L $ZnSO_4$, 0.075 mg/L $CoCl_2$, 0.075 mg/L H_3BO_3, 0.09 mg/L KI and 0.06 mg/L $Na_2MoO_4\cdot 2H_2O$) were introduced into the reactor. The reactor was operated at 30°C with an HRT and SRT of 12h and 11d, respectively. The pH was controlled at 7.00-7.60, with DO levels maintained at 0.8-1.2 mg/L during the aerobic phase.

S2.3 Sequencing batch reactor operation (SBR3) [3]**.** The SBR3 with a working volume of 4.5 L was inoculated with activated sludge collected from the same WWTP as SBR1 for the enrichment of *Ca.* Accumulibacter for lactate and succinate utilization test. The SBR was operated with 6 h cycles, including a slow-feeding phase (60 min), an anaerobic phase (20 min), an aerobic phase (180 min), and a settling/decant phase (100 min). Acetate was used as sole carbon source for the reactor. In each cycle, 2.5 L of synthetic wastewater was fed into the reactor, formulated based on the same composition as that used for SBR1, but adjusted to contain 100 mg TOC/L sodium acetate and 20 mg/L PO_4^{3-}-P, resulting in a TOC/P molar ratio of 15:1. The HRT and SRT in the reactor were 12 h and 15 d, respectively. The pH, DO, and temperature control systems were configured in the same manner as described for SBR1.

S2.4 Sequencing batch reactor operation (SBR4 and SBR5). SBR4 and SBR5 with a working volume of 5.0 L was inoculated with activated sludge collected from the same full-scale WWTP as SBR1 for the enrichment of *Ca.* Accumulibacter for MAG recovery. Both reactors followed 6-h operational cycles, consisting of a feeding/anaerobic phase (80 min), an aerobic phase (180 min), and a settling/decant phase (100 min). The influent wastewater was prepared using the same composition as described for SBR3. The same HRT (12 h) was applied for both reactors with different feeding strategy and SRT. SBR4 employed a slow-feeding phase (60 min) with an SRT of 25 days. SBR5 adopted a fast-feeding phase (12 min) with an SRT of 15 days. The pH, DO, and temperature control conditions were configured in the same manner as described for SBR1.

S2. 5 Bioreactor 6. The Bioreactor 6 was a 2.5 L lab-scale membrane bioreactor (MBR), inoculated with activated sludge from the same WWTP as SBR1 for the enrichment of hydroxylamine-tolerant and hydroxylamine-degrading bacterial community. The reactor was continuously fed with synthetic wastewater, with an aeration rate maintained at 9.0 L/min. The synthetic wastewater medium contained the following basal components: 0.149 g/L $NH_2OH{\cdot}HCl$, 0.5 g/L $MgSO_4{\cdot}7H_2O$, 0.107 g/L $CaCl_2{\cdot}2H_2O$, and 0.02 g/L KH_2PO_4. Trace element solutions were supplemented per liter as follows: 1 mL of solution I (containing 10 g/L EDTA-2Na and 15 g/L $FeSO_4{\cdot}7H_2O$), 0.1 mL of solution II (including 4.3 g/L $ZnSO_4{\cdot}7H_2O$, 2.4 g/L $CoCl_2{\cdot}6H_2O$, 9.9 g/L $MnCl_2{\cdot}4H_2O$, 2.5 g/L $CuSO_4{\cdot}5H_2O$, and 1.9 g/L $NiCl_2{\cdot}6H_2O$), and 0.1 mL of solution III (with 2.2 g/L $NaMoO_4{\cdot}2H_2O$ and 0.14 g/L H_3BO_3). The HRT was set at 12 h, and the DO concentration was maintained at 5-6 mg/L.

S3. Full-scale studies and batch tests

S3.1 Full-cycle study on the PAO enrichment cultures in SBR1. Anaerobic-aerobic full-scale studies were performed on Day 149 on the enrichment culture in SBR1 at the reactor's original operation regime (i.e. with 45 mgTOC/L glucose as the sole carbon source, as mentioned in S2.1). Water and activated sludge samples were collected at 0, 5, 15, 30, 45, 60, 90, 120, 135, 150, 165, 180, 210, 240, 270, 300, 330, and 350 minutes for PO_4^{3-}-P, TOC analyses. PO_4^{3-}-P concentrations were determined following the Standard Methods [4]. TOC was measured using a TOC analyzer (Shimadzu, Japan). Activated sludge samples were collected just before the start of the full cycle (0 min), at 5 min (anaerobic phase), 15 min (anaerobic phase), 135 min (anaerobic phase), and 150 min (aerobic phase), snap-frozen in liquid N_2, and stored at -80°C before DNA/RNA extraction for metagenomic and metatranscriptomic analyses.

S3.2 Anaerobic P release/carbon uptake test with amino acids by using the *Ca.* Accumulibacter enrichment culture in SBR2 [2]. For anaerobic P release tests, fresh activated sludge was collected from the reactor at the end of the aerobic phase. The activated sludge was diluted with the effluent from the reactor to a mixed liquor suspended solids (MLSS) concentration of 1.0 g/L, and the solution pH was adjusted to 7.0 using 0.1 M HCl or 0.1 M NaOH. Fifty milliliters of diluted activated sludge were added into 50 mL culture bottles and sealed. Anaerobic conditions were induced by N_2 gas purging for 15 min before the addition of different carbon sources to different

bottles. Twenty α-amino acids (all of them are L-isomers except for glycine) were tested and compared with acetate. Each carbon source was added to individual culture bottles to a final TOC concentration of 250 mg/L. In all batch tests performed in this study, carbon sources were added as one pulse at the beginning of each experiment to facilitate the determination of carbon uptake kinetics and stoichiometry. The culture bottles were incubated for 5 h in an incubator (Infors HT, Bottmingen, Switzerland), operated at 180 rpm and 30 °C. Water samples were collected every hour by passing them immediately through 0.45 μm sterile filters for PO_4^{3-}-P analysis. One culture bottle with activated sludge and no carbon source addition served as a control. The experiments were done in triplicate.

S3.3 Full-cycle study on the *Ca.* Accumulibacter enrichment culture in SBR3 [3]. To evaluate the ability and related metabolism of *Ca.* Accumulibacter in using lactate and succinate for EBPR, anaerobic-aerobic full cycle studies were performed on Day 783 in SBR3 with lactate and succinate as carbon sources (each at 40 mg/L) respectively. The full cycle contains an 80 min anaerobic stage (including a 60-min slow-feeding stage and a 20-min anaerobic stage afterwards) and a 180 min aerobic stage. Water and activated sludge samples was collected at 5, 15, 30, 45, 60, 80, 105, 120, 150, 180, 210 and 260 min for TOC, PO_4^{3-}-P, PHA and glycogen analyses. To further investigate whether *act*P is involved in the transport of lactate and acetate, a mixed-carbon condition was tested in which both acetate and lactate were added at 40 mg/L each (total 80 mg/L). This treatment was compared with conditions using either substrate alone. All other operational parameters were identical to the single-carbon-source experiments. TOC and PO_4^{3-}-P measurements followed the same analytical procedures as described in S3.1. PHA analyses were performed according to Oehmen et al. (2005) [5], using a Trace Ultra Gas Chromatography (equipped with a DB-5MS column, 30 m ×0.25 mm, Agilent Technology, USA) coupled to a DSQ II mass spectrometer (Thermo Scientific, USA). Glycogen analyses were carried out according to the method described by Kristiansen et al.[6]. Lyophilized activated sludge was resuspended in 5 mL of 0.9 M HCl and digested at 100 °C for 5 h. Glucose equivalents in the supernatant were quantified using a HPLC (E2695, Waters, US) equipped with a HyREZ XP column (Dionex, Thermo Fisher, Denmark). Activated sludge samples were also collected just before the start of the full cycle (0 min), at 5 min (anaerobic phase), 30 min (anaerobic phase), 105 min (anaerobic phase), and 120 min (aerobic phase), snap-frozen in liquid N_2, and stored at -80 °C before RNA extraction for metatranscriptomic analysis.

S4. 16S rRNA gene amplicon sequencing, metagenomic and metatranscriptomic analysis

S4.1 DNA extract and sequencing of enrichment cultures. Activated sludge samples from the enrichment bioreactors were used for metagenomic analysis. Genomic DNA were extracted using the OMEGA Soil DNA Kit (D5625-01) (Omega Bio-Tek, GA, USA), following the manufacturer's instructions, and stored at -80°C prior to further analyses. The quantity and quality of the extracted DNA were measured using a Qubit™ 4 Fluorometer (with WiFi: Q33238; Qubit™ Assay Tubes: Q32856; Qubit™

1X dsDNA HS Assay Kit: Q33231) and 1% agarose gel electrophoresis, respectively. Genomic library was constructed following the Illumina TruSeq DNA Sample Preparation Guide. Paired-end (PE150) sequencing was performed using the Illumina NovaSeq platform. Raw reads were submitted to NCBI under the BioProject No. PRJNA1191061 and PRJNA771771.

S4.2 16S rRNA gene amplicon sequencing of enrichment cultures. For the microbial community analysis of the enrichment culture in SBR2, 2 ml of mixed liquor were collected on Day 152 and Day 166 from the end of the aerobic stage and stored at -80°C before DNA extraction. Genomic DNA was extracted using the Fast DNATM 2 mL SPIN Kit for Soil samples (MP Biomedicals, CA, USA), following the optimized protocol for activated sludge.1 Bacterial 16S rRNA gene amplicon sequencing was performed, targeting the V1-V3 region (primer set: 27F AGAGTTTGATCCTGGCTCAG and 534R ATTACCGCGGCTGCTGG). PCR amplification was carried out in a 25-μl PCR matrix containing 10 ng of genomic DNA, 400 nM dNTPs, 1.5 mM $MgSO_4$, 2 mU Platinum R Taq DNA polymerase high fidelity, 1× Platinum R High Fidelity buffer (Thermo Fisher Scientific, MA, USA) and a pair of barcoded library adaptors (400 nM), with a thermo cycler setting of initial denaturation at 95°C for 2 min, 30 cycles of 95°C for 20 s, 56°C for 30 s, 72°C for 60 s, and final elongation at 72°C for 5 min. All PCR reactions were run in duplicate and pooled afterwards. The amplicon libraries were purified using the Agencourt R AMpure XP bead protocol (Beckmann Coulter, CA, USA) with 1.8 bead solution/PCR solution ratio. Based on library concentrations and calculated amplicon sizes, the samples were pooled in equimolar concentrations. The library pool was sequenced on a MiSeq (Illumina, CA, US) using a MiSeq Reagent kit v3 (2×300 paired end). Pre-processing of all amplicon libraries was performed according to Albertsen et al. Taxonomy was assigned using MiDAS v.1.20.

S4.3 Metatranscriptomic analysis. Activated sludge samples collected during the full-cycle experiments (see Section S3.1 and S3.3) were subjected to metatranscriptomic analysis. Total RNA was extracted using the RNA PowerSoil® Total RNA Isolation Kit (Omega Bio-Tek, GA, USA). The quality and quantity of the extracted RNA were measured using 1.5% agarose gel electrophoresis and UV spectrophotometer, respectively. cDNA library was constructed using a TruSeq Standard mRNA LT Sample Prep Kit (Illumina, CA, USA). The quality and quantity of the library were measured using Agilent Bioanalyzer and Promega QuantiFluor, respectively. 10 ng RNA was loaded for each sample. Illumina NovaSeq sequencing was performed at a read-length of 150 bp paired-end. fastp [1] and SortMeRNA [7] were used to remove adaptation sequences and ribosomal ribonucleic acids (rRNAs). Filtered reads were mapped to the corresponding MAGs using BBMap version 38.96 [8] and were normalized to Reads Per Kb-CDSs Per Million Mapped Reads (RPKM). Raw reads were submitted to NCBI under the BioProject No. PRJNA1191061 (SBR1 samples) and PRJNA771771 (SBR3 samples).

S5. Dynamic constraint-based modeling of EBPR cycles

To simulate the dynamic metabolic behavior of *Ca.* Accumulibacter in EBPR systems, we developed a constraint-based dynamic flux balance analysis (cFBA) framework that tracks selected metabolites over time while assuming quasi–steady-state flux distributions at each discrete time step based on previously studies [9, 10]. The genome-scale metabolic models were first reconstructed via gapseq and exported in the SBML format. The cFBA simulations covered a complete 5h EBPR cycle, consisting of a 1.5h anaerobic phase and a 3.5h aerobic phase, with a temporal resolution of 0.2 h (25 steps per cycle). At each time step, a Linear Programming (LP) problem was formulated and solved using COBRApy (v0.26.3), with a defined objective function and updated constraints based on dynamic pool sizes. A subset of key metabolites was explicitly treated as in non-steady states, including extracellular substrate (acetate or other carbon sources), glycogen, PHB, PH2MV, polyphosphate (PolyP), inorganic phosphate (Pi), CO_2, and biomass. These pools were numerically integrated using a forward Euler scheme, where the updated amount of each metabolite was calculated based on its net production/consumption flux and the time step length. During the anaerobic phase, oxygen uptake and all electron transport chain (ETC) reactions were constrained to zero, while a finite dose of carbon sources (2.0 mmol · gDW^{-1}) was provided and consumed at a controlled rate to mimic depletion within the first 1 h. Polyphosphate (polyP) hydrolysis (rxn15388) was enabled and its synthesis (rxn06117) was blocked, and the LP objective was set to minimize the total weighted flux (parsimonious solution), favoring energy-efficient pathways. In contrast, during the aerobic phase, oxygen uptake was enabled. External carbon uptake was disabled. polyP synthesis was re-enabled with a small positive lower bound, and the objective function was switched to biomass maximization with pFBA, under a constant non-growth-associated ATP maintenance demand of 0.398 mmol · gDW^{-1} · h^{-1}. Each dynamic metabolite pool was associated with pseudo exchange reactions (e.g., STIN_PolyP, STOUT_PolyP) that allowed controlled accumulation and utilization. Their flux bounds were updated based on the available pool size at each step to maintain mass balance. These parameter choices and simulation logic were informed by the constraint-based dynamic EBPR models developed by Páez-Watson et al. [9, 10], ensuring biological relevance and methodological consistency. The resultant time-resolved fluxes and metabolite levels successfully captured canonical PAO behaviors, including acetate uptake and polyP degradation during the anaerobic phase, and subsequent polyP resynthesis and biomass growth in the aerobic phase. All model files, SBMLs, simulation scripts, and time-course outputs are publicly accessible via GitHub (https://github.com/Xiaojing-Xie/FBA_PAOS) and Science Data Bank (https://doi.org/10.57760/sciencedb.18043).

S6. Viral sequence identification and dereplication

For all the metagenomic data mentioned above, assembled contigs were used for putative viral contig identification using four different viral identification methods (ViralVerify v1.158 [11], VIBRANT v1.2.159 [12], VirSorter2 v 2.2.4 [13] and DeepVirfinder [14] (scores > 0.85 and p values < 0.05). The identified putative viral contigs were filtered using geNomad [15], which were then merged for host

contamination removal and completeness estimation using CheckV [16]. Viral contigs with lengths <5 kb was discarded. After filtering out host contaminants, the predicted viral sequences were clustered into vOTUs following standard guidelines at 95% identity and 85% alignment fraction of the smallest scaffolds based on the scripts provided in CheckV.

S7. Species etymology

The following 21 novel species of *Ca.* Accumulibacter are proposed, each named according to Latin grammatical convention and in compliance with the International Code of Nomenclature of Prokaryotes. Names derive from personal honors, geographical origins, institutional associations, or ecological symbolism.

"*Candidatus* Accumulibacter wuertzii" (wuer'tzi.i.) is named in honor of Prof. Stefan Wuertz, for his contributions to environmental microbiology and wastewater biotechnology.

"*Candidatus* Accumulibacter nuwaii" (nu'wa.i.i.) is named after Nüwa, a mythological figure in Chinese culture symbolizing ecological restoration and harmony.

"*Candidatus* Accumulibacter heboyii" (he.bo'y.i.i.) is named after Hebo, the river god of Chinese mythology, representing freshwater ecological origin.

"*Candidatus* Accumulibacter brasiliensis" (bra.si.li.en'sis) refers to Brazil, where this species was observed at high abundance in full-scale wastewater treatment systems.

"*Candidatus* Accumulibacter varunius" (va.ru'ni.us.) is derived from Varuna, a Vedic deity associated with water and ecological adaptability.

"*Candidatus* Accumulibacter poseidonii" (po.sei.do'ni.i.) is named after Poseidon, the Greek god of the sea, indicating aquatic adaptation.

"*Candidatus* Accumulibacter sirenis" (si.re'nis) is derived from the mythological sirens of Greek mythology, reflecting ecological niche specialization.

"*Candidatus* Accumulibacter undinae" (un.di'nae) is named after Undina, a Latinized water spirit, indicating adaptation to aqueous ecosystems.

"*Candidatus* Accumulibacter tetysi" (te'ty.si) is named after Tethys, the Greek Titaness associated with primordial waters, metaphorically referencing ancient liquid-phase environments.

"*Candidatus* Accumulibacter aquariius" (a.qua.ri'i.us) is derived from Latin *aquarius*, meaning water-bearer, to reflect its aquatic lifestyle.

"*Candidatus* Accumulibacter houyii" (hou'y.i.i) is named in honor of Houyi, a mythological archer symbolizing environmental control and balance.

"*Candidatus* Accumulibacter mcmahonii" (mac.ma.ho'ni.i) is named in honor of Prof. Katherine D. McMahon, for foundational research on *Ca.* Accumulibacter and EBPR microbiomes.

"*Candidatus* Accumulibacter loosdrechtii" (loos.drech'ti.i) is named in honor of Prof. Mark C. M. van Loosdrecht, for his pioneering work in wastewater microbial ecology.

"*Candidatus* Accumulibacter xiamensis" (ksi.a.men'sis) refers to the city of Xiamen, China, where this species was first recovered.

"***Candidatus* Accumulibacter epflensis"** (ep.len'sis) is named after EPFL (École Polytechnique Fédérale de Lausanne), the institution where this MAG was first identified.
"***Candidatus* Accumulibacter zhurongii"** (zhu.rong'i.i) is named after Zhurong, the Chinese god of fire, symbolizing energy transformation and microbial metabolism.
"***Candidatus* Accumulibacter zhangtongii"** (zhang.ton'gii) is named in honor of Prof. Tong Zhang, recognizing his contributions to environmental metagenomics.
"***Candidatus* Accumulibacter hkuensis"** (h.k.u.en'sis) refers to the University of Hong Kong (HKU), the institution where this MAG was first reported.
"***Candidatus* Accumulibacter netherlandicus"** (neer.lan'di.cus) refers to the Netherlands, indicating the country of origin for this species.
"***Candidatus* Accumulibacter helmoltzianus"** (hel.molt.zi'a.nus) is named after the Helmholtz Centre for Environmental Research (UFZ), Germany, for its contributions to wastewater microbiome research.
"***Candidatus* Accumulibacter nielsenii"** (nie.lse'ni) is named in honor of Prof. Per H. Nielsen, for his pioneering work in activated sludge microbial ecology.

Supplementary Tables

Table S1. Different phylogenetic taxonomies of species in *Ca*. Accumulibacter

Name	Clade	species	Completeness, %	Contamination, %
UW4	IA	*Ca*. A. regalis	92.9	3.37
MAG-203	IA	*Ca*. A. regalis	95.27	1.01
MAG13	IA	*Ca*. A. regalis	100	0.95
CANDO1	IA	*Ca*. A. regalis	91.67	0.52
bin32	IA	*Ca*. A. regalis	98.81	2.09
bin163	IA	*Ca*. A. regalis	100	1.92
AMR_MDS_5487	IA	*Ca*. A. regalis	100	0.27
ACC007	IA	*Ca*. A. regalis	98.1	4.39
ACC005	IA	*Ca*. A. regalis	97.85	2.17
UW8-POB	IA	*Ca*. A. regalis	99.84	1.69
AMR_MDS_5463	IB	*Ca*. A. helmoltzianus	95.91	0.71
HKU-1	IB	*Ca*. A. adiacens	90.38	2.54
UWLDOIC	IC	*Ca*. A. meliphilus	95.24	1.1
UW14	IC	*Ca*. A. meliphilus	95.24	4.58
SBR_S	IC	*Ca*. A. delftensis	97.34	0.66
mtI13	IC	*Ca*. A. delftensis	99.05	1.06
ACC012	IC	*Ca*. A. delftensis	95.87	4.63
MAG3	ID	*Ca*. A. netherlandicus	98.89	1.44
HK-STAS-PROT-102	ID	*Ca*. A. hkuensis	91.77	0.74
HK-STAS-PROT-103	IF	*Ca*. A. zhangtongii	90.24	0.03
Bin140	IF	*Ca*. A. zhangtongii	99.49	0.03
Bin208	IG	*Ca*. A. zhurongii	99.42	0.42
AMR_MDS_5503	IG	*Ca*. A. zhurongii	94.4	0.16
ACC003	IG	*Ca*. A. epflensis	98.57	0.35
Bin45	IIA	*Ca*. A. xiamensis	99.05	3.36
MAG-197	IIA	*Ca*. A. loosdrechtii	93.9	4.08
MAG33	IIA	*Ca*. A. loosdrechtii	96.54	0.84
AALB	IIA	*Ca*. A. aalborgensis	99.52	0.06
UW1	IIA	*Ca*. A. phosphatis	100	0.24
UW16	IIB	*Ca*. A. propinquus	97.62	0.77
UW10-POB	IIB	*Ca*. A. propinquus	90.71	2.01
mtII8	IIB	*Ca*. A. propinquus	97.86	3.93
MAXAC027	IIB	*Ca*. A. propinquus	97.67	4
BATAC726	IIB	*Ca*. A. propinquus	90.17	2.33
BAT3C415	IIB	*Ca*. A. propinquus	95.26	0.98
UBA5574	IIC	*Ca*. A. vicinus	93.65	0.24
SK01	IIC	*Ca*. A. vicinus	93.73	1.19
UW17	IIC	*Ca*. A. contiguus	97.29	1.22
SBR_L	IIC	*Ca*. A. contiguus	96.85	0.98

GWASMC-A37	IIC	*Ca*. A. contiguus	98.03	0.66
Bin28	IIC	*Ca*. A. contiguus	91.82	1.06
UW6	IIC	*Ca*. A. cognatus	98.57	2.46
UW11-POB	IIC	*Ca*. A. cognatus	92.96	1.59
SSA1	IIC	*Ca*. A. cognatus	99.05	1.11
SK02	IIC	*Ca*. A. cognatus	97.62	1.59
SCUT-5	IIC	*Ca*. A. cognatus	98.19	1.22
SCUT-2	IIC	*Ca*. A. cognatus	98.1	1.11
SCUT-1	IIC	*Ca*. A. cognatus	96.98	1.59
SCELSE-8	IIC	*Ca*. A. cognatus	95.45	2.06
SCELSE-2	IIC	*Ca*. A. cognatus	95.4	1.11
MAG-201	IIC	*Ca*. A. cognatus	92.72	1.59
HKU-2	IIC	*Ca*. A. cognatus	92.29	2.86
GWASMC-A24	IIC	*Ca*. A. cognatus	94.72	3.41
Bin142	IIC	*Ca*. A. cognatus	92.74	0.95
AMR_MDS_0334	IIC	*Ca*. A. cognatus	94.16	1.53
UW18	IIC	*Ca*. A. mcmahonii	98	2.54
GWASMC-A32	IIC	*Ca*. A. mcmahonii	98	0.88
GWASMC-A31	IIC	*Ca*. A. mcmahonii	95.38	1.48
GWASMC-A30	IIC	*Ca*. A. mcmahonii	97.46	0.71
GWASMC-A27	IIC	*Ca*. A. mcmahonii	96.09	1.67
GWASMC-A1	IIC	*Ca*. A. mcmahonii	98.71	0.32
MAG-200	IID	*Ca*. A. proximus	94.33	1.01
BATAC285	IID	*Ca*. A. proximus	96.67	3.63
UW12-POB	IID	*Ca*. A. necessarius	98.1	0.98
SCELSE-6	IIF	Ca. A. houyii	96.22	0.48
MAG-198	IIF	*Ca*. A. tetysi	92.93	3.09
GWASMC-A25	IIF	*Ca*. A. tetysi	97.46	3.05
UW21	IIF	*Ca*. A. aquariius	98.1	0.03
SK12	IIF	*Ca*. A. aquariius	92.6	0.05
SSB1	IIF	*Ca*. A. similis	99.05	0.03
SCELSE-1	IIF	*Ca*. A. similis	95.24	0.6
bin12	IIF	*Ca*. A. similis	96.69	0.16
UBA2327	IIF	*Ca*. A. iunctus	92.43	0.29
UW13-POB	IIF	*Ca*. A. conexus	97.14	4.39
GWASMC-A29	IIF	*Ca*. A. conexus	98.02	0.66
BAT3C720	IIG	*Ca*. A. affinis	93.81	0.98
SCELSE-7	IIH	*Ca*. A. tropicus	98.1	0.61
GWASMC-A16	IIH	*Ca*. A. tropicus	96.3	2.72
Bin228	III	*Ca*. A. sirenis	96.09	0.24
GWASMC-A8	III	*Ca*. A. sirenis	95.59	2.39
GWASMC-A6	III	*Ca*. A. sirenis	94.6	0.77
GWASMC-A49	III	*Ca*. A. sirenis	91.37	4.16
GWASMC-A40	III	*Ca*. A. sirenis	96.52	0.21

GWASMC-A4	III	*Ca.* A. sirenis	95.51	1.79
GWASMC-A38	III	*Ca.* A. sirenis	96.11	0.27
GWASMC-A23	III	*Ca.* A. sirenis	96.11	0.33
GWASMC-A22	III	*Ca.* A. sirenis	94.11	1.62
GWASMC-A20	III	*Ca.* A. sirenis	90.6	2.48
GWASMC-A19	III	*Ca.* A. sirenis	93.81	1.14
GWASMC-A18	III	*Ca.* A. sirenis	92.16	1.13
GWASMC-A17	III	*Ca.* A. sirenis	95.49	2.94
GWASMC-A14	III	*Ca.* A. sirenis	93.9	1.55
GWASMC-A13	III	*Ca.* A. sirenis	94.31	0.3
GWASMC-A12	III	*Ca.* A. sirenis	94.05	1.94
GWASMC-A10	III	*Ca.* A. sirenis	96.05	1.87
AMR_MDS_0713	III	*Ca.* A. sirenis	93.21	3.77
AMR_MDS_0653	III	*Ca.* A. sirenis	94.01	1.16
AMR_MDS_0563	III	*Ca.* A. sirenis	96.4	1.22
AMR_MDS_0347	III	*Ca.* A. sirenis	90.33	0.9
SCELSE-14	III	*Ca.* A. undinae	93.62	1.79
UW26	IIJ	*Ca.* A. jenkinsii	90.03	1.02
UW25	IIJ	*Ca.* A. jenkinsii	97.25	1.05
SCELSE-9	IIK	*Ca.* A. torridus	97.83	0.19
GWASMC-A39	IIL	*Ca.* A. nielsenii	96.43	1.66
GWASMC-A36	IV-A	*Ca.* A. poseidonii	96.46	0.48
GWASMC-A35	IV-A	*Ca.* A. poseidonii	96.94	0.12
GWASMC-A33	IV-A	*Ca.* A. poseidonii	97.59	0.48
GWASMC-A2	IV-A	*Ca.* A. poseidonii	97.59	0.48
AMR_MDS_3893	IV-A	*Ca.* A. poseidonii	99.02	0
GWASMC-A28	IV-B	*Ca.* A. varunius	99.53	3.35
GWASMC-A26	IV-B	*Ca.* A. brasiliensis	92.1	0.16
GWASMC-A43	IV-B	*Ca.* A. brasiliensis	97.16	0
GWASMC-A42	IV-B	*Ca.* A. brasiliensis	94.79	0
GWASMC-A34	IV-B	*Ca.* A. brasiliensis	98.58	0.12
MAG-196	IV-C	*Ca.* A. nuwaii	95.52	0.48
SCUT-4	IV-C	*Ca.* A. nuwaii	99.2	2.1
SCUT-8	IV-C	*Ca.* A. heboyii	95	0.95
SCUT-6	IV-C	*Ca.* A. heboyii	92.06	2.39
SCUT-7	IV-C	*Ca.* A. wuertzii	98.13	3.43
SCELSE-11	IV-C	*Ca.* A. wuertzii	97.62	0.48
GWASMC-A9	IV-C	*Ca.* A. wuertzii	96.43	1.35
GWASMC-A7	IV-C	*Ca.* A. wuertzii	100	3.22
GWASMC-A50	IV-C	*Ca.* A. wuertzii	94.86	2.96
GWASMC-A5	IV-C	*Ca.* A. wuertzii	99.26	4.13
GWASMC-A48	IV-C	*Ca.* A. wuertzii	94.92	2.18
GWASMC-A47	IV-C	*Ca.* A. wuertzii	92.4	4.43
GWASMC-A46	IV-C	*Ca.* A. wuertzii	91.34	1.97

GWASMC-A45	IV-C	*Ca*. A. wuertzii	91.6	2.54
GWASMC-A44	IV-C	*Ca*. A. wuertzii	90.07	2.24
GWASMC-A41	IV-C	*Ca*. A. wuertzii	99.05	0.95
GWASMC-A3	IV-C	*Ca*. A. wuertzii	98.1	1.73
GWASMC-A21	IV-C	*Ca*. A. wuertzii	96.19	2.33
GWASMC-A15	IV-C	*Ca*. A. wuertzii	95.08	0.48
GWASMC-A11	IV-C	*Ca*. A. wuertzii	97.38	4.23
AMR_MDS_0859	IV-C	*Ca*. A. wuertzii	97.38	0.53
AMR_MDS_0531	IV-C	*Ca*. A. wuertzii	93.97	0.48

Table S2. Pairwise nucleotide identity ranges of *ppk1* genes within and between *Ca.* Accumulibacter clades.

**Similarity (%)**	**I**	**II**	**III**	**IV**
**I**	85.65-99.86			
**II**	82.58-87.33	81.11-99.95		
**III**	82.93-87.30	81.90-88.12	92.63-99.95	
**IV**	81.10-84.90	80.65-85.04	82.56-84.79	85.72-99.95

Table S3. Mean and median relative abundances (%) of *Ca.* Accumulibacter and *Ca.* Phosphoribacter based on 340 WWTP samples. "A_" denotes *Ca.* Accumulibacter, and "P_" denotes *Ca.* Phosphoribacter.

Continent	A_mean	A_median	P_mean	P_median
Africa	3.83	1.41	0.41	0.26
Asia	0.85	0.28	0.75	0.40
Europe	1.09	0.85	4.96	2.24
North America	2.51	1.25	0.60	0.10
Oceania	1.55	1.13	0.39	0.10
South America	0.40	0.09	0.33	0.03

Table S4. Mean and median relative abundances (%) of *Ca.* Accumulibacter and *Ca.* Phosphoribacter based on 237 EBPR-associated WWTP samples. "A_" denotes *Ca.* Accumulibacter, and "P_" denotes *Ca.* Phosphoribacter.

Continent	A_mean	A_median	P_mean	P_median
Africa	3.83	1.41	0.41	0.26
Asia	0.83	0.26	0.85	0.45
Europe	1.11	0.85	5.14	2.49
North America	4.13	3.03	1.05	0.27
Oceania	1.32	0.86	0.56	0.37
South America	1.10	1.46	0.06	0.00

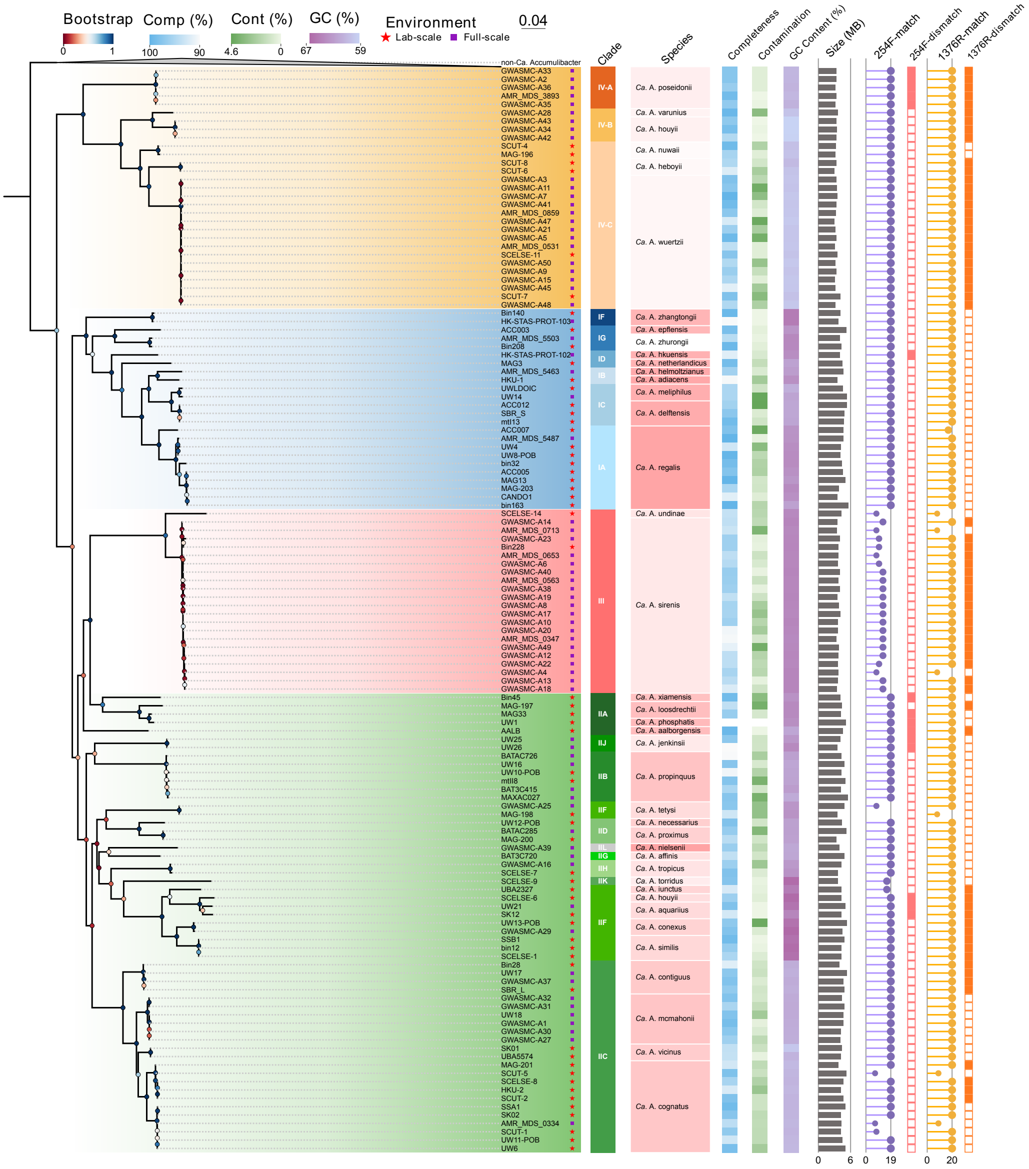

Fig. S1 Phylogenomic tree of *Ca.* Accumulibacter based on *ppk1* gene sequences, annotated with genome features and primer binding mismatches. Clade classifications are indicated alongside. Associated genome features are shown in the metadata panels, including genome completeness (%), contamination (%), genome size (Mb), GC content (%), and mismatch rates against the commonly used 254F (forward) and 1376R (reverse) primers. Environments of genome recovery (lab-scale vs. full-scale reactors) are also indicated.

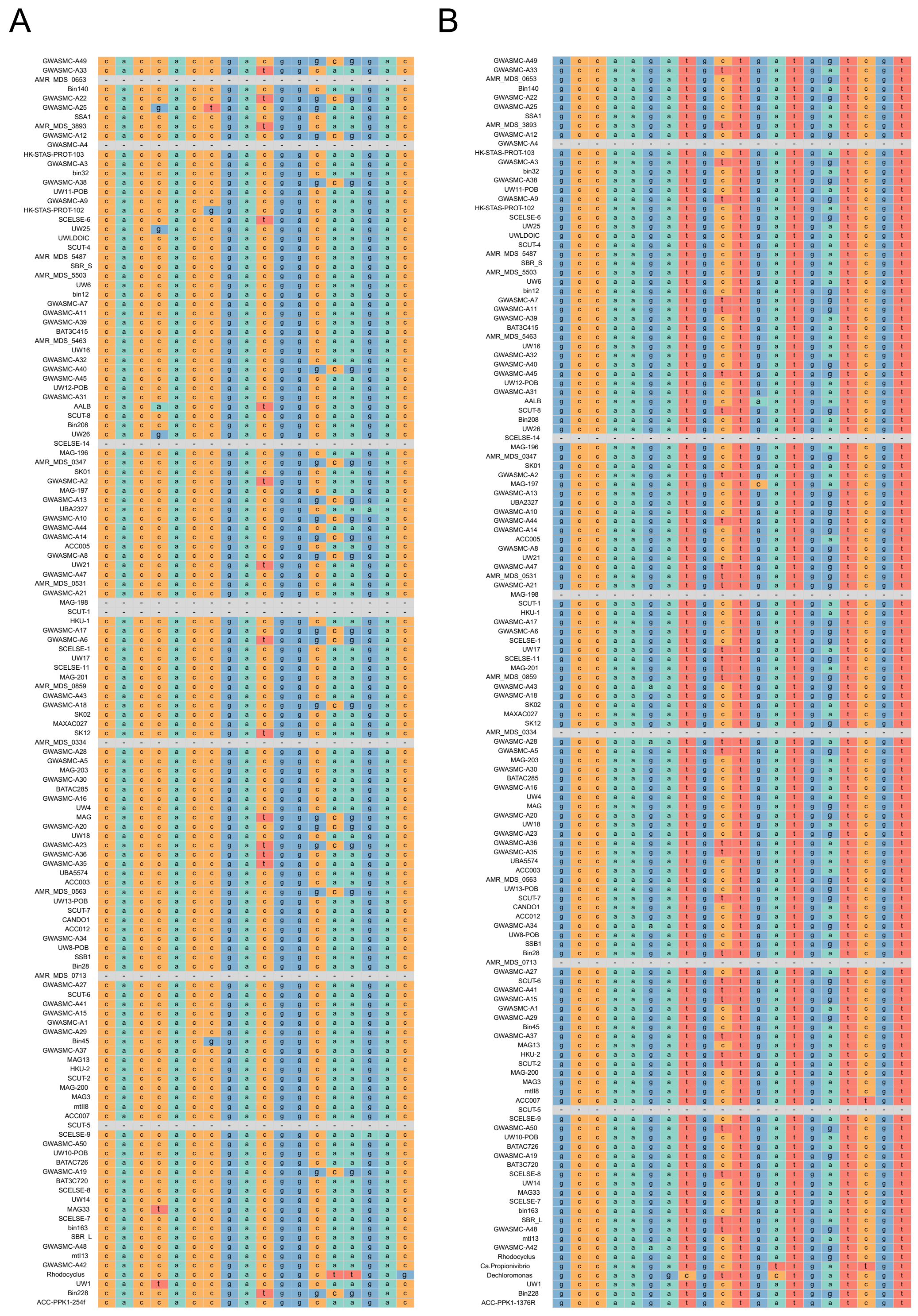

Fig. S2. Sequence alignment results of *Ca*. Accumulibacter *ppk1* genes against primer regions of the widely used 254F/1376R primer set.(A) Alignment of *ppk1* sequences with the forward primer 254F;(B) Alignment with the reverse primer 1376R.

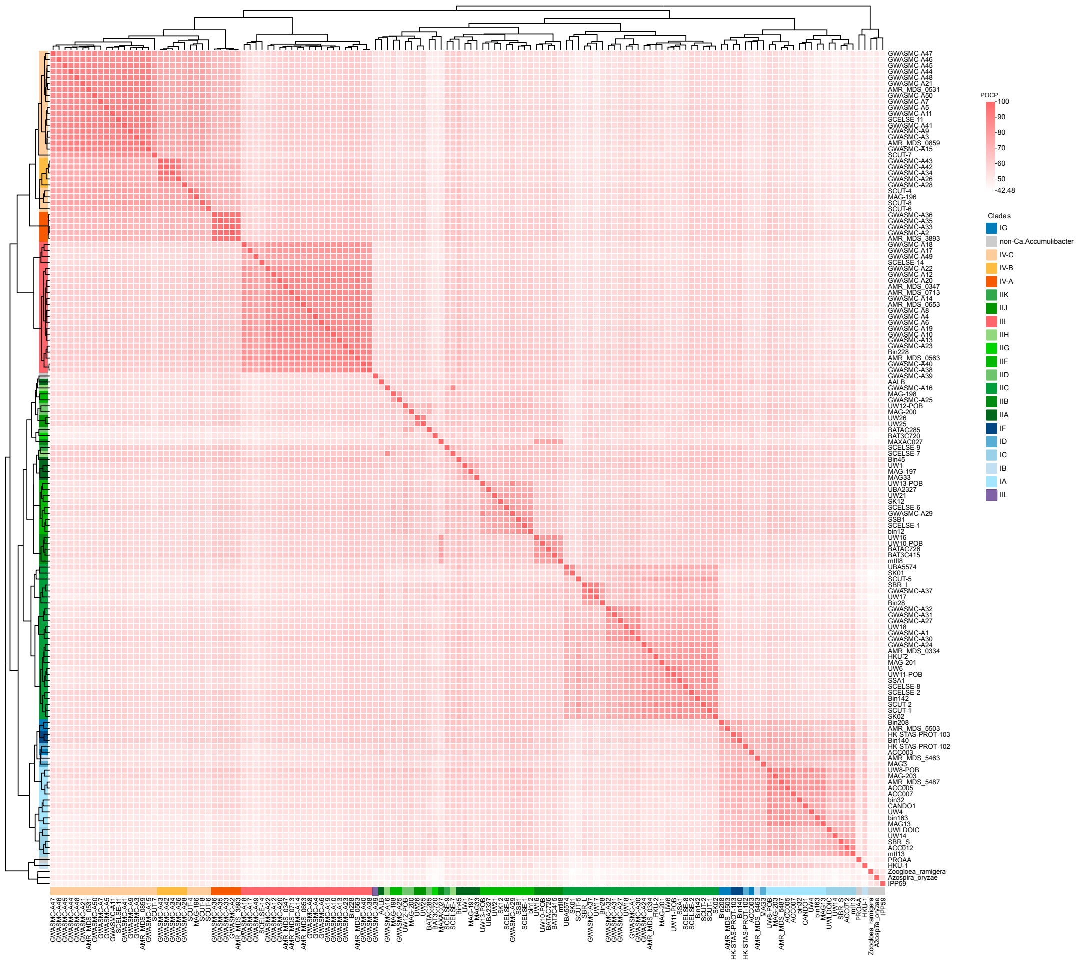

Fig. S3. Pairwise percentage of conserved proteins (POCP) similarity among *Ca.* Accumulibacter MAGs.

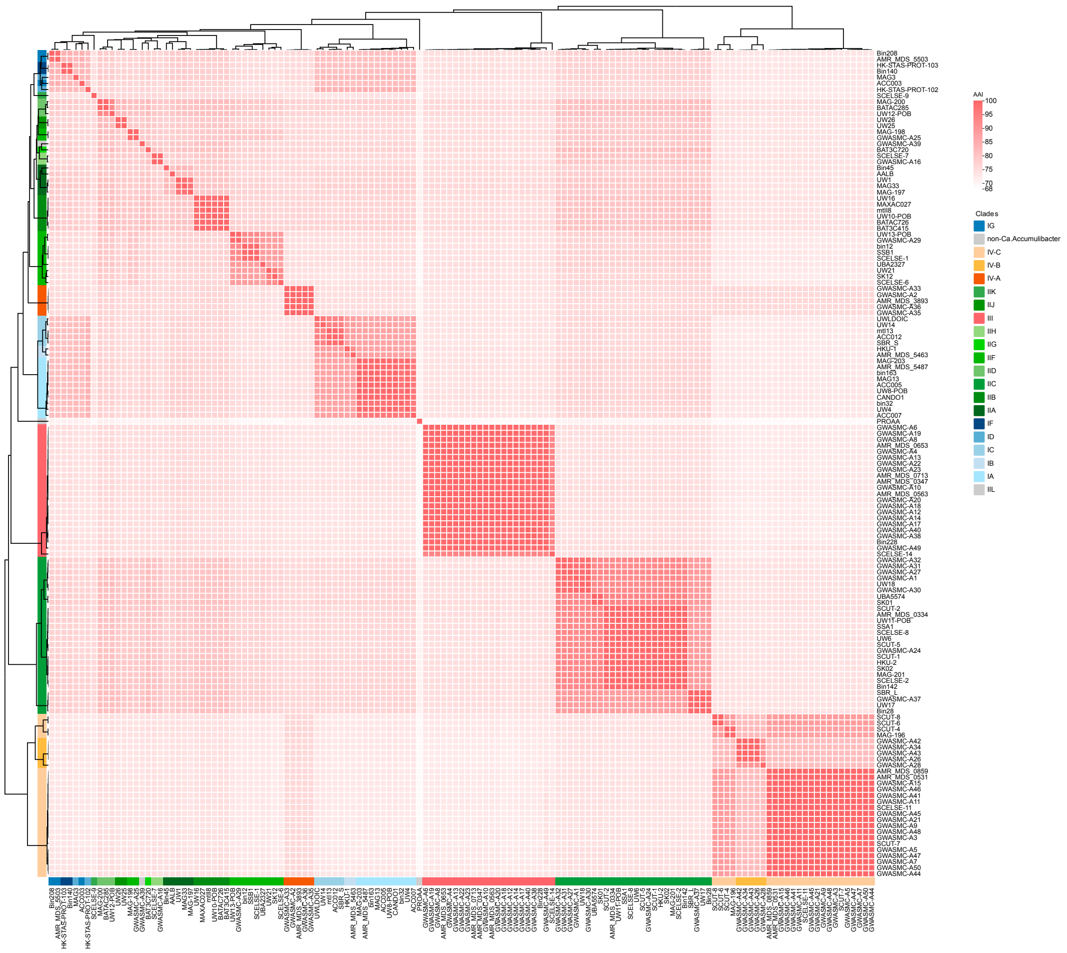

Fig. S4. Pairwise average amino acid identity (AAI) among *Ca*. Accumulibacter MAGs.

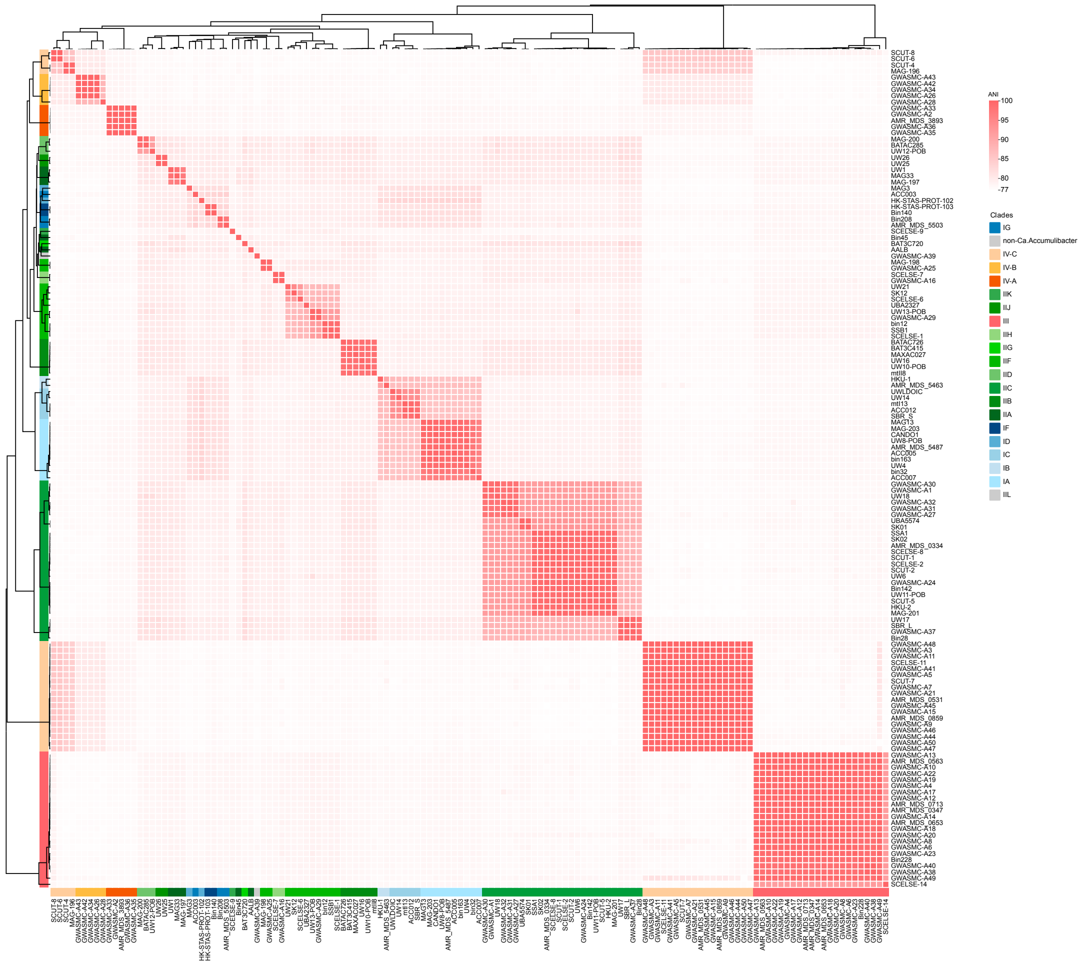

Fig. S5. Pairwise average nucleotide identity (ANI) among *Ca*. Accumulibacter MAGs.

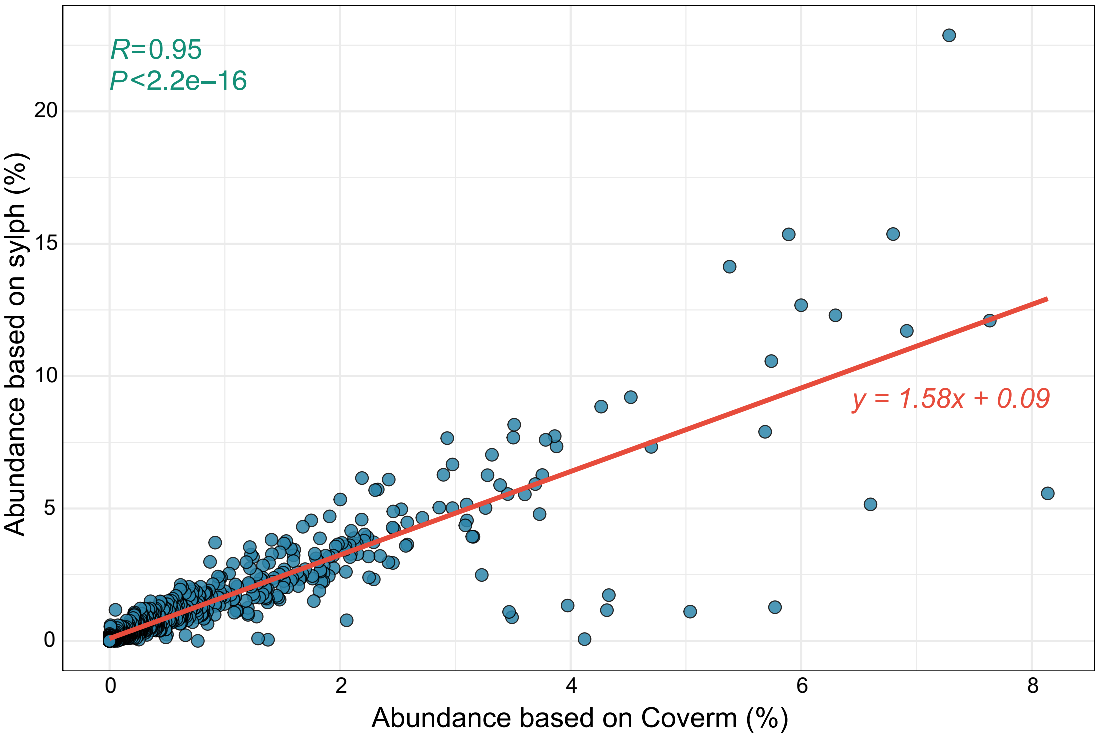

Fig. S6. Correlation between two relative abundance estimation methods. Each point represents one genome/sample pair. The red line indicates the fitted linear regression (y = 1.58x + 0.09). The high Pearson correlation coefficient ($r = 0.95$, $p < 2.2e\text{-}16$) indicates strong consistency between the two methods.

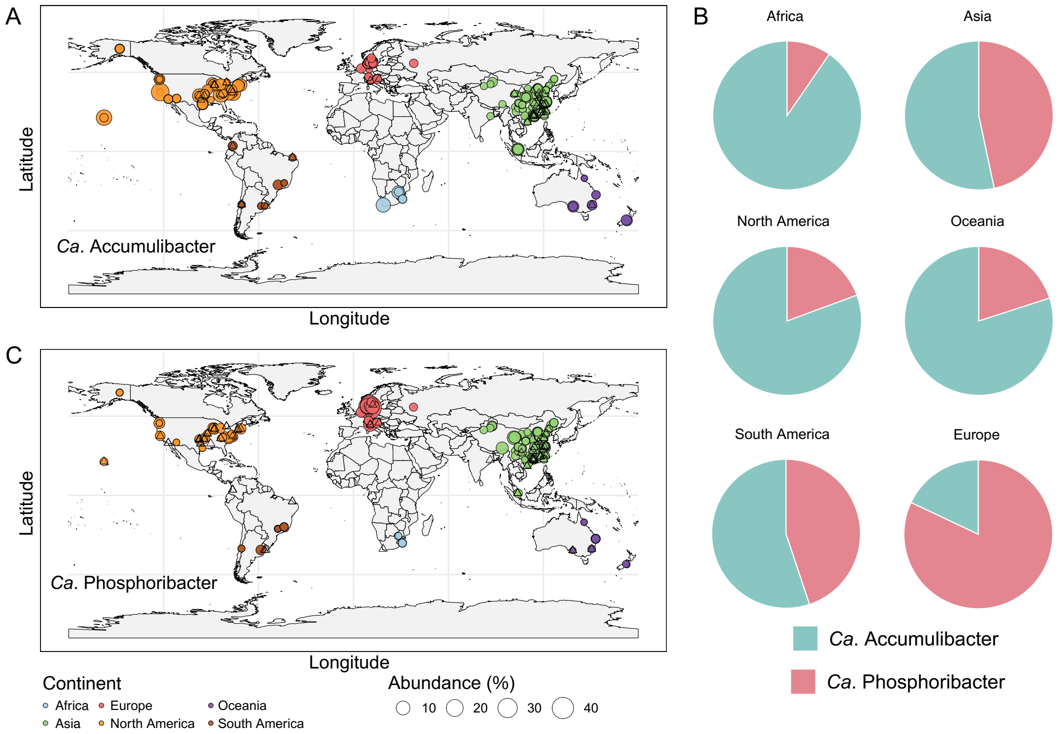

Fig. S7. Global distribution and genus-level relative abundance of *Ca.* Phosphoribacter and *Ca.* Accumulibacter in 340 distinct WWTPs. Geographic distribution of *Ca.* Phosphoribacter (A) and *Ca.* Accumulibacter (B), with circle sizes indicating genus-level relative abundance (calculated using Sylph) across 340 WWTPs. Colors denote continents. (C) Genus-level relative abundance ratio of *Ca.* Accumulibacter (green) and *Ca.* Phosphoribacter (pink) across six continents. For each continent, the relative abundances of the two genera were summed and normalized to 100% for intuitive comparison. The results show that *Ca.* Accumulibacter are dominant in Africa, Asia, North America, Oceania, and South America, whereas *Ca.* Phosphoribacter show higher relative abundances in Europe.

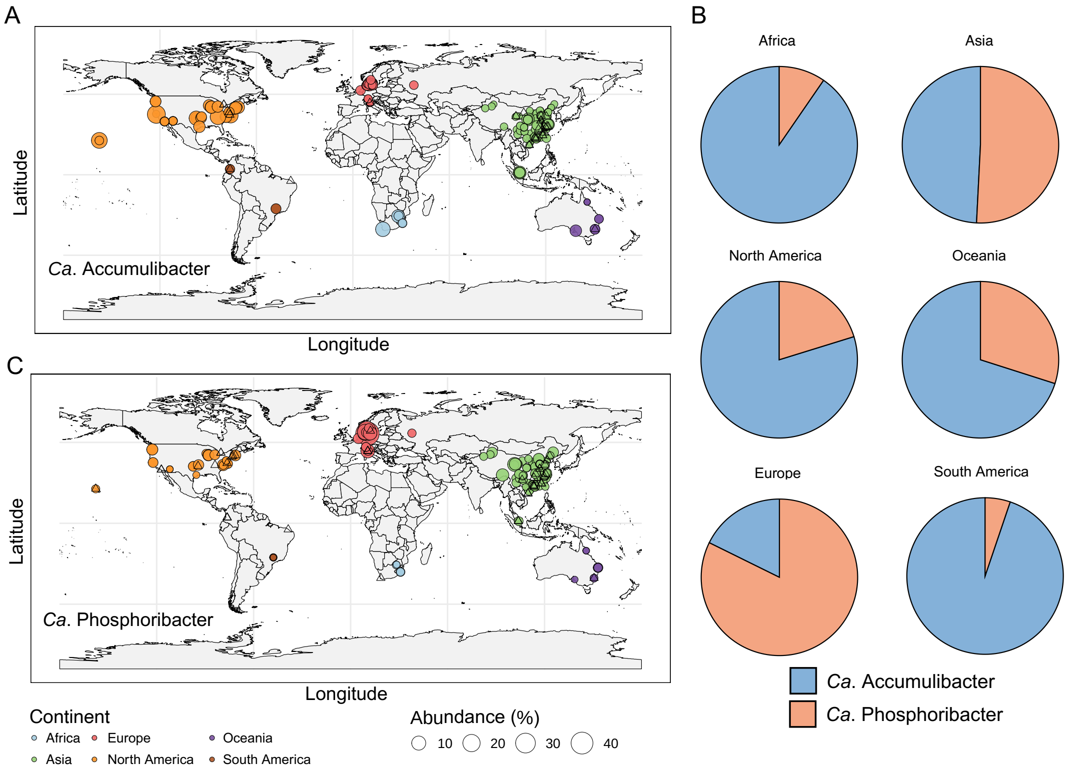

Fig. S8. Global distribution and genus-level relative abundance of *Ca.* Phosphoribacter and *Ca*. Accumulibacter in 237 EBPR-associated WWTPs. Geographic distribution of *Ca.* Phosphoribacter (A) and *Ca.* Accumulibacter (B), with circle sizes indicating genus-level relative abundance (calculated using Sylph) across 237 WWTPs. Colors denote continents. (C) Genus-level relative abundance ratio of *Ca.* Accumulibacter (blue) and *Ca.* Phosphoribacter (orange) across six continents. For each continent, the relative abundances of the two genera were summed and normalized to 100% for intuitive comparison. The results show that *Ca.* Accumulibacter are dominant in Africa, North America, Oceania, and South America, whereas *Ca.* Phosphoribacter show higher relative abundances in Europe and Asia.

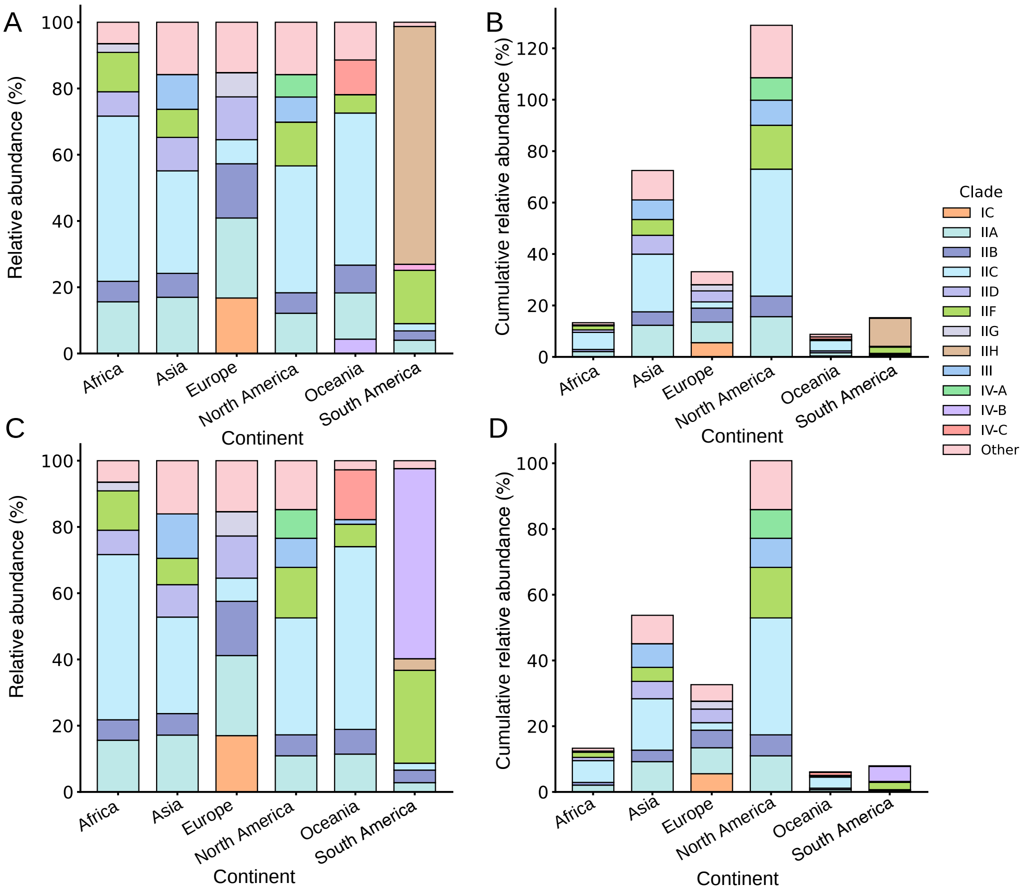

Fig S9. Clade-level distribution of *Ca.* Accumulibacter across continents based on 340 WWTPs. (A) Relative abundance (%) and (B) cumulative relative abundance across all WWTPs. (C) Relative abundance (%) and (D) cumulative relative abundance in confirmed EBPR WWTPs.

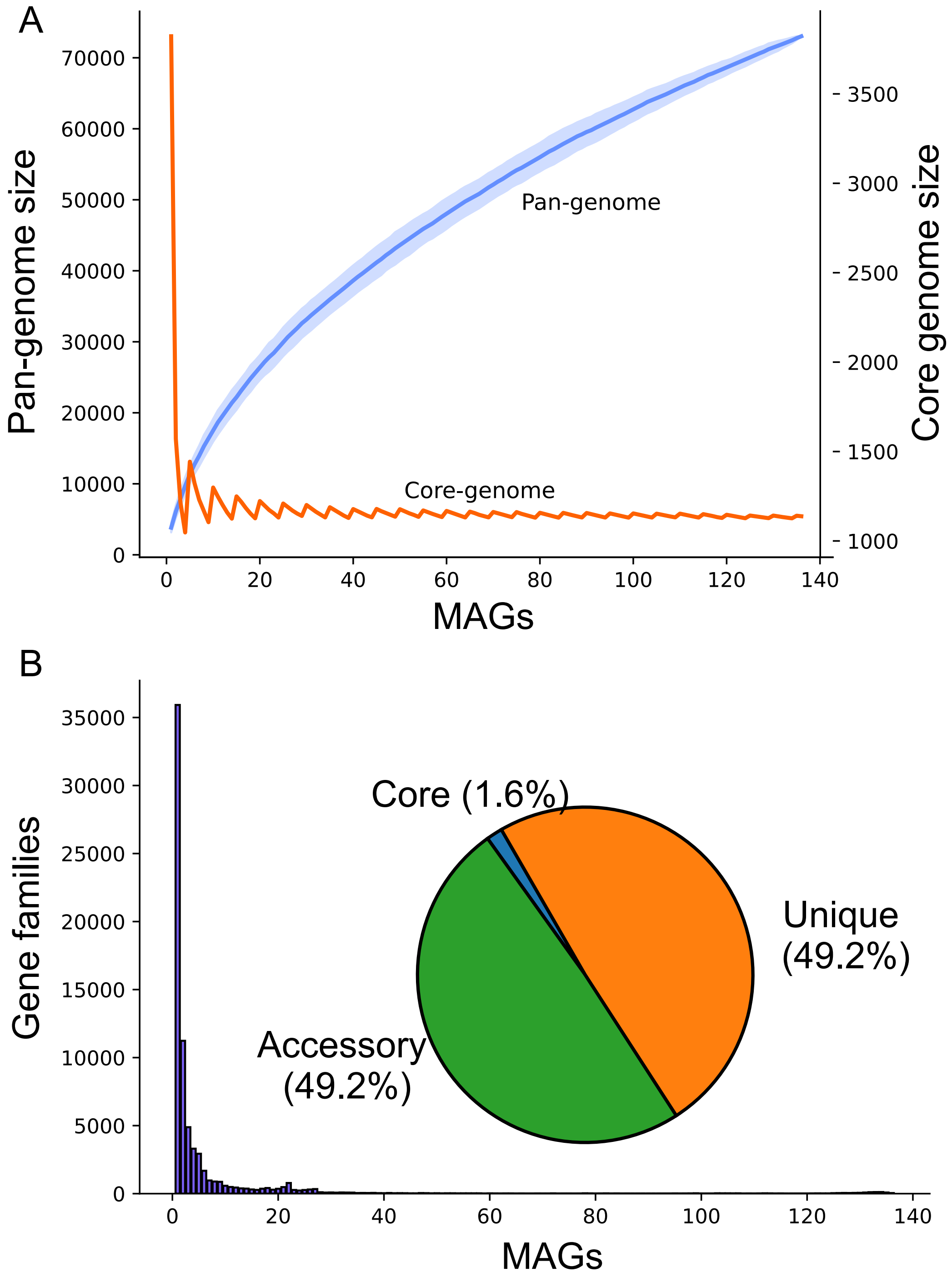

Fig. S10. Overview of the pan-genome landscape of *Ca*. Accumulibacter. (A) Cumulative pan-genome and core-genome curves based on 136 *Ca.* Accumulibacter MAGs. The left y-axis represents the cumulative number of gene families in the pan-genome and core genome, while the right y-axis denotes the percentage of core gene families (defined as those present in ≥80% of genomes). (B) Distribution of gene family frequencies across the dataset. The histogram illustrates the number of gene families present in different numbers of genomes. Core, accessory (moderately conserved), and unique gene families are shown as proportions of the total.

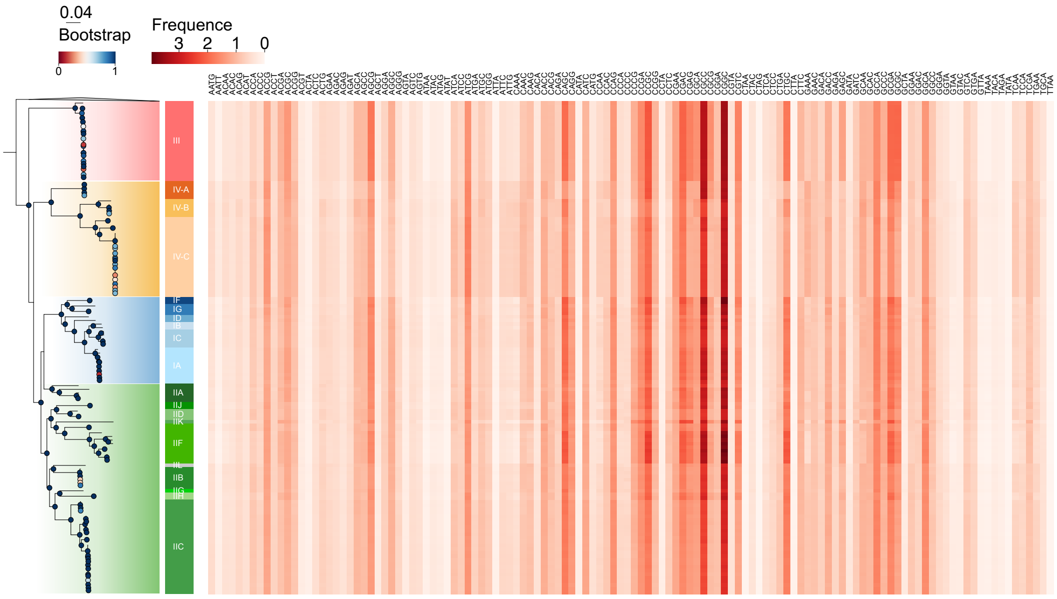

Fig. S11. Hierarchical clustering of 136 *Ca*. Accumulibacter MAGs based on tetranucleotide (4-mer) frequency profiles.

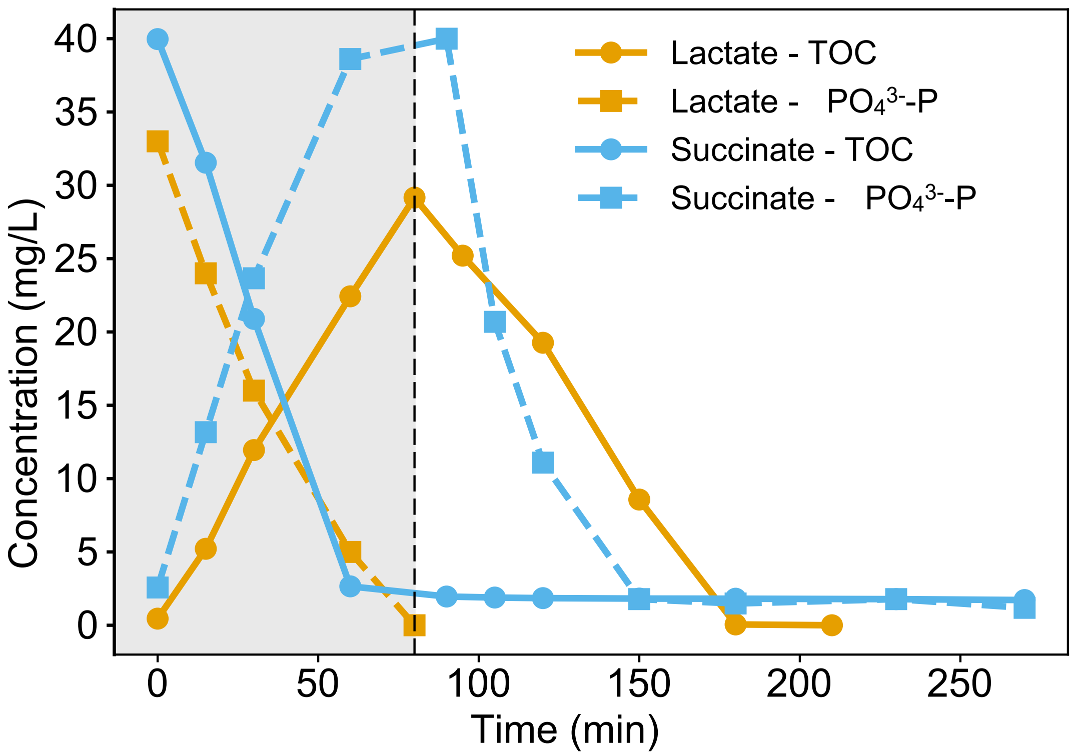

Fig. S12. Changes in PO_4^{3-}-P and TOC concentrations during EBPR cycles with lactate or succinate as a sole carbon source. Experiments were performed with the *Ca.* Accumulibacter enrichment culture obtained in SBR3 (S3.3). Dashed lines indicate transitions between anaerobic and aerobic phases.

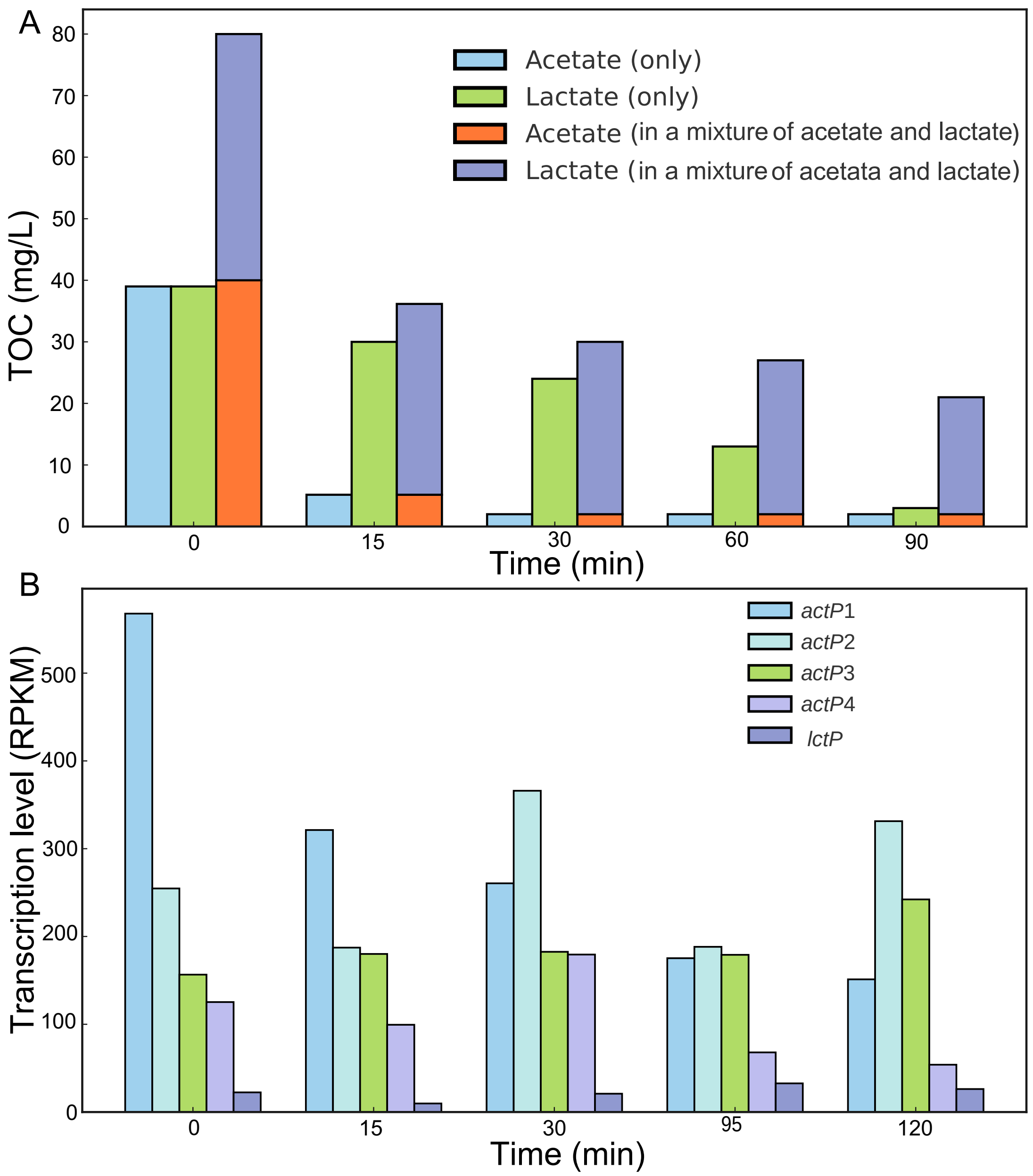

Fig. S13. (A) A comparison of the uptake profiles of lactate when it was fed separately or together with acetate as revealed via high-performance liquid chromatography analyses. (B) Transcriptional dynamics of acetate permease gene (*actP*, 4 copies) and the lactate permease gene (*lctP*, 1 copy) in *Ca*. Accumulibacter cognatus SCUT-2 recovered from SBR3 (S3.3) under lactate-fed conditions.

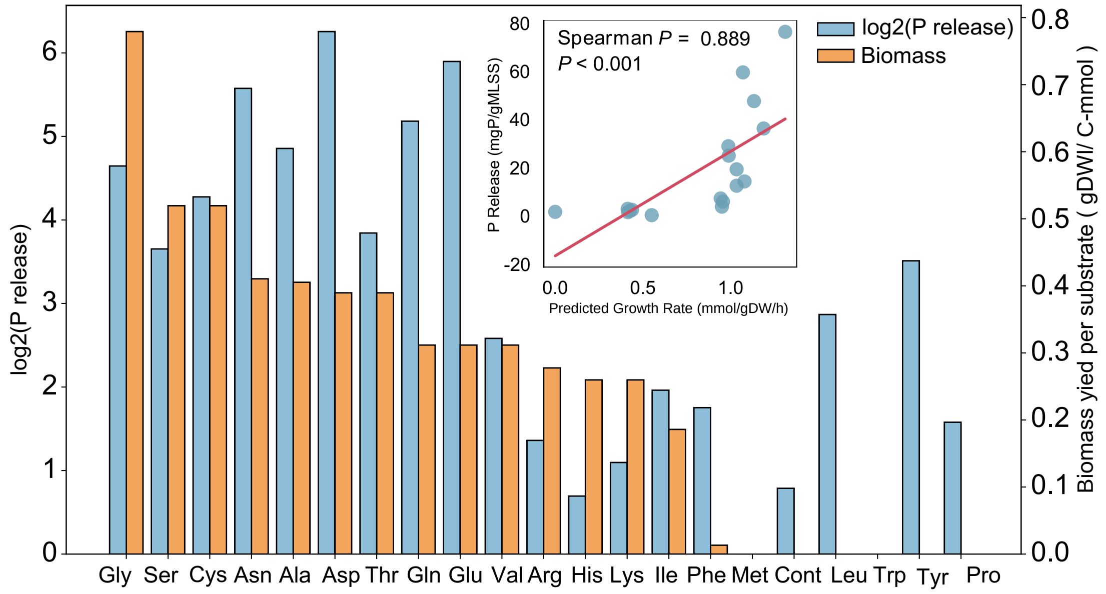

Fig. S14. Anaerobic phosphate release after 5 hours and model-predicted growth rates of *Ca.* Accumulibacter similis SCELSE-1 (recovered in SBR2, S3.2) following the addition of different amino acids. A positive correlation is observed between phosphate release and predicted growth.

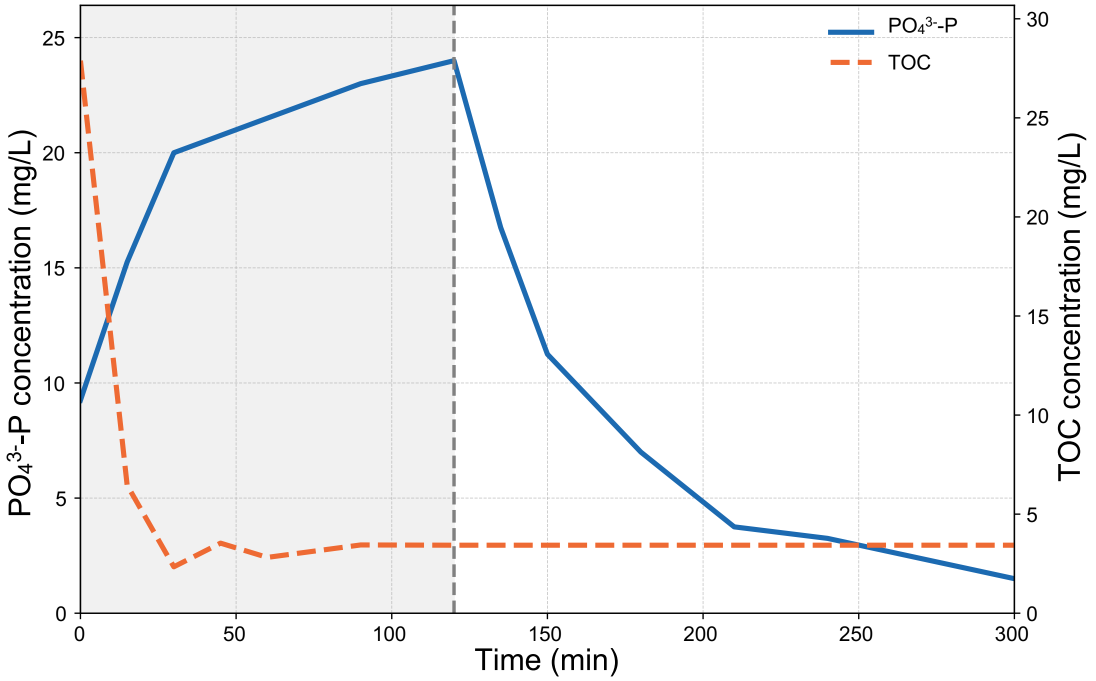

Fig. S15. Time series of PO_4^{3-}-P and TOC (glucose as a sole carbon source) concentrations during a typical EBPR cycle in SBR1 (S3.1). The vertical dashed line at 120 min demarcates the transition from anaerobic to aerobic conditions.

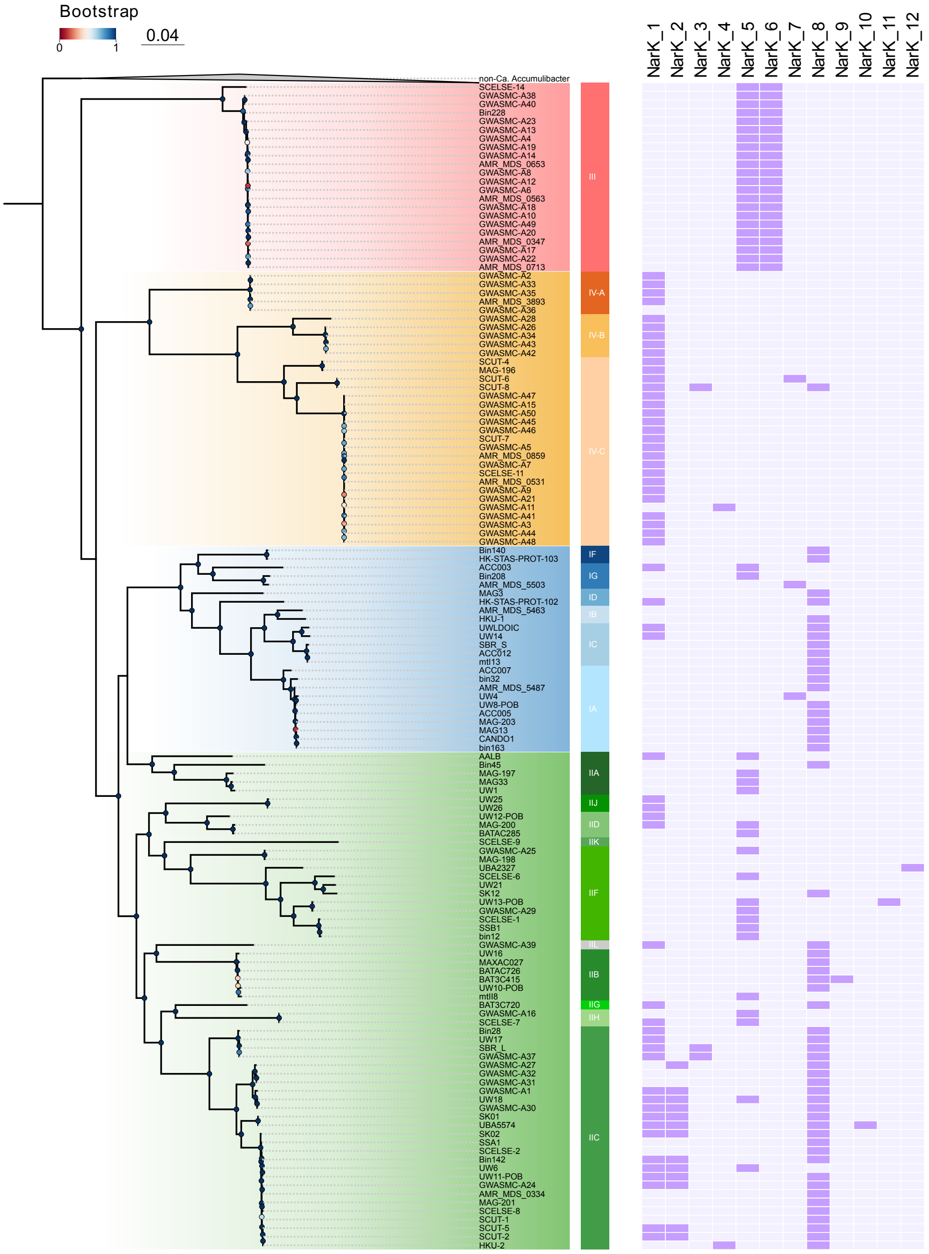

Fig. S16. Distribution of NarK transporter homologs across *Ca*. Accumulibacter MAGs. Twelve types of NarK-like proteins were identified based on sequence homology and phylogenetic reconstruction, each exhibiting distinct lineage-specific gain or loss patterns.

Supplementary Data

Dataset S1. Summary of metagenome metadata and *Ca.* Accumulibacter relative abundance profiles across 828 activated sludge microbiomes. Sheet 1- Detailed metadata for 828 metagenome samples in this study, including the NCBI identifiers, sampling locations (country and continent), as well as latitude and longitude coordinates. Sheet 2- The relative abundance of representative *Ca*. Accumulibacter MAGs, clustered at 95% ANI, across the global WWTPs. Relative abundance was estimated based on read recruitment using CoverM in the MAG mode with ≥95% identity and ≥75% alignment thresholds. Sheet 3- For each continent (based on all 340 WWTPs), the table shows the cumulative relative abundance and proportion of each species (clustered at 95% ANI) in the Ca. Accumulibacter community, along with the number of samples analyzed. Sheet 4- For each continent (based on 237 EBPR-associated WWTPs), the table shows the cumulative relative abundance and proportion of each species (clustered at 95% ANI) in the Ca. Accumulibacter community, along with the number of samples analyzed.

Dataset S2. Pan-genome analysis of *Ca.* Accumulibacter. Sheet 1- all homologous gene clusters (pan-genes) identified across the *Ca*. Accumulibacter MAGs, including the representative gene for each cluster. Sheet 2- a matrix summarizing the number of genes assigned to each homologous gene cluster across all individual *Ca*. Accumulibacter MAGs. Sheet 3- the details of clade- and species-specific gene clusters. Lineage-specific genes were defined as gene clusters present exclusively in a given clade or species, and absent from all other clades and species, indicating potential functional differentiation or ecological specialization.

Dataset S3. Community-level amino acid flux and co-occurrence patterns associated with *Ca.* Accumulibacter. Sheet 1- the predicted amino acid secretion fluxes for MAGs co-recovered with *Ca*. Accumulibacter from WWTPs. Flux values >0 indicate the capacity for net amino acid secretion, based on genome-scale metabolic modeling. Sheet 2- the summarize of 216 MAGs predicted to secrete one or more amino acids, including their taxonomic classifications and the specific amino acids they can produce. Sheet 3- the list of microbial species that show co-occurrence with *Ca*. Accumulibacter across global WWTPs, defined as having a Spearman correlation coefficient >0.3 based on relative abundance profiles.

Dataset S4. Microbial communities and gene transcription associated with carbon source utilization by *Ca.* Accumulibacter. Sheet 1- Microbial communities of the enrichment culture used for lactate and succinate utilization tests, based on metagenomic sequencing. Sheet 2- Microbial community composition of the enrichment culture for amino acids utilization batch tests, based on 16S rRNA sequencing. Sheet 3- MAGs recovered and their relative abundances in the enrichment culture with glucose as a carbon source, based on metagenomic analysis. Sheet 4- Transcriptomic profiles of *Propionicimonas* ((MAG SBR1_531.68,) recovered under glucose as the carbon source. Sheet 5- Transcriptomic profiles of *Arachnia* (MAG SBR1_531.17) recovered under glucose as the carbon source.

Dataset S5. Antiviral defense systems and CRISPR-based virus–host interactions in *Ca.* Accumulibacter. Sheet 1- the summarize of the number of predicted major

categories of antiviral defense systems (DSs) encoded by each *Ca*. Accumulibacter MAG. Sheet 2- the detail of the number of subtypes of DSs identified per MAG. Sheet 3- CRISPR spacer matches between *Ca.* Accumulibacter genomes and viral sequences recovered from WWTPs, representing potential virus–host interactions inferred from CRISPR-based targeting.

Reference

1. Chen S, Zhou Y, Chen Y, Gu J. Fastp: An ultra-fast all-in-one fastq preprocessor. *Bioinformatics*. 2018; **34**: i884-i90 https://doi.org/10.1093/bioinformatics/bty560
2. Qiu G, Liu X, Saw NMMT *et al.* Metabolic traits of candidatus accumulibacter clade iif strain scelse-1 using amino acids as carbon sources for enhanced biological phosphorus removal. *Environ. Sci. Technol.* 2020; **54**: 2448-58 https://doi.org/10.1021/acs.est.9b02901
3. Chen L, Wei G, Zhang Y *et al.* Candidatus accumulibacter use fermentation products for enhanced biological phosphorus removal. *Water Res*. 2023; **246**: 120713 https://doi.org/10.1016/j.watres.2023.120713
4. Association APH. *Standard methods for the examination of water and wastewater*, vol. 6: Am. J Public Health, 1926.
5. Oehmen A, Zeng RJ, Yuan Z, Keller J. Anaerobic metabolism of propionate by polyphosphate-accumulating organisms in enhanced biological phosphorus removal systems. *Biotechnol. Bioengineering*. 2005; **91**: 43-53 https://doi.org/10.1002/bit.20480
6. Kristiansen R, Nguyen HTT, Saunders AM *et al.* A metabolic model for members of the genus tetrasphaera involved in enhanced biological phosphorus removal. *ISME J*. 2013;**7**:543-54 https://doi.org/10.1038/ismej.2012.136
7. Kopylova E, Noé L, Touzet H. Sortmerna: Fast and accurate filtering of ribosomal rnas in metatranscriptomic data. *Bioinformatics*. 2012; **28**: 3211-17 https://doi.org/10.1093/bioinformatics/bts611
8. Bushnell B. Bbmap: A fast, accurate, splice-aware aligner. In: *Conference: 9th Annual Genomics of Energy & Environment Meeting, Walnut Creek, CA, March 17-20, 2014, United States*. Abstract Alignment of reads is one of the primary computational tasks in bioinformatics. Of paramount importance to resequencing, alignment is also crucial to other areas - quality control, scaffolding, string-graph assembly, homology detection, assembly evaluation, error-correction, expression quantification, and even as a tool to evaluate other tools. An optimal aligner would greatly improve virtually any sequencing process, but optimal alignment is prohibitively expensive for gigabases of data. Here, we will present BBMap [1], a fast splice-aware aligner for short and long reads. We will demonstrate that BBMap has superior speed, sensitivity, and specificity to alternative high-throughput aligners bowtie2 [2], bwa [3], smalt, [4] GSNAP [5], and BLASR [6]. p. Medium: ED. DE-AC02-05CH11231 2016-04-08, 2014.

9. Páez-Watson T, van Loosdrecht MCM, Wahl SA. From metagenomes to metabolism: Systematically assessing the metabolic flux feasibilities for "*Candidatus* Accumulibacter" species during anaerobic substrate uptake. *Water Res*. 2024; **250**: 121028 https://doi.org/10.1016/j.watres.2023.121028
10. Páez-Watson T, Jansens C, van Loosdrecht MCM, Roy S. Co-substrate utilisation in "*Candidatus* Accumulibacter" enhances metabolic fitness in dynamic environments. *Water Res*. 2025; **287**: 124401 https://doi.org/10.1016/j.watres.2025.124401
11. Antipov D, Raiko M, Lapidus A, Pevzner PA. Metaviralspades: Assembly of viruses from metagenomic data. *Bioinformatics*. 2020; **36**: 4126-29 https://doi.org/10.1093/bioinformatics/btaa490
12. Kieft K, Zhou Z, Anantharaman K. Vibrant: Automated recovery, annotation and curation of microbial viruses, and evaluation of viral community function from genomic sequences. *Microbiome*. 2020; **8**: 90 https://doi.org/10.1186/s40168-020-00867-0
13. Guo J, Bolduc B, Zayed AA *et al.* Virsorter2: A multi-classifier, expert-guided approach to detect diverse DNA and rna viruses. *Microbiome*. 2021; **9**: 37 https://doi.org/10.1186/s40168-020-00990-y
14. Ren J, Song K, Deng C *et al.* Identifying viruses from metagenomic data using deep learning. *Quant. Biol*. 2020; **8**: 64-77
15. Camargo AP, Roux S, Schulz F *et al.* Identification of mobile genetic elements with genomad. *Nat. Biotechnol*. 2024; **42**: 1303-12 https://doi.org/10.1038/s41587-023-01953-y
16. Camargo AP, Nayfach S, Chen IA *et al.* Img/vr v4: An expanded database of uncultivated virus genomes within a framework of extensive functional, taxonomic, and ecological metadata. *Nucleic Acids Res*.2023; **51**: D733-d43 https://doi.org/10.1093/nar/gkac1037
